# Charge Density and Magnetic Properties in Cobalt(II) Single‐Molecule Magnets: Impact of Ligand Substitution

**DOI:** 10.1002/cphc.70404

**Published:** 2026-05-11

**Authors:** Katharina Rachuy, Paula Stark, Regine Herbst‐Irmer, Dietmar Stalke

**Affiliations:** ^1^ Institut für Anorganische Chemie Georg‐August‐University Göttingen Göttingen Germany

**Keywords:** charge density, high‐resolution X‐ray diffraction, single‐molecule magnet *d*‐metal, superconducting Quantum interference device

## Abstract

The cobalt(II) complex [Co{(NSiMe_3_)_2_SPh}_2_] (**2_Co**) was synthesized and compared to the known analog [Co{(N^*t*^Bu)_2_SPh}_2_] (**1_Co**) in order to assess the effect of ligand substitution on charge density and magnetic properties. High‐resolution X‐ray diffraction with multipole refinement and quantum theory of atoms in molecules analysis indicated small but noticeable differences in bond character, with **2_Co** showing a tendency toward slightly enhanced ionic contributions. Bader charge analysis suggested a somewhat higher electron density at cobalt in **2_Co**, although experimental *d*‐orbital populations displayed inconsistencies and only partly agreed with ab initio calculations. Magnetic measurements of **2_Co** confirmed significant anisotropy (*D* = –138.9 cm^−1^) and field‐dependent relaxation dynamics influenced by quantum tunneling, with properties comparable to, but in some respects marginally improved over **1_Co**. These results highlight that subtle ligand changes influence bonding and relaxation behavior, though the overall differences between the two complexes remain modest. Benchmarking of experimental and computational approaches clearly needs more data from either method.

## Graphical abstract

Two Co(II) complexes with slight differences in their SN_2_ ligands were studied by magnetic measurements, experimental charge density determination, and computational methods to determine the cobalt *d*‐orbital population and the effect on different single‐molecule magnet properties.

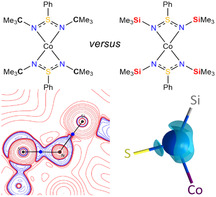

## 1 Introduction

In recent years, polyimidosulfinates and ‐sulfonates [1, 2, 3, 4, 5] turned out to be suitable *N*,*N*‐chelating ligands for single‐molecule magnets (SMMs) [6, 7, 8, 9, 10, 11, 12] in *d*‐metal [13, 14, 15] and f‐metal [13, 16, 17] chemistry. They are coordination complexes that exhibit slow relaxation of magnetization due to bistable spin ground states separated by an energy barrier [18]. Unlike conventional bulk magnets, which lose their long‐range magnetic ordering when reduced to molecular dimensions owing to the absence of magnetic domains, SMMs retain their magnetic properties at the molecular scale. This makes them highly attractive for potential applications in spintronics and high‐density data storage [19, 20, 21, 22]. In 2021, we reported on a series of tetrahedral distorted Co^2+^ complexes with SN‐based ligands and found that a N–Co–N bite angle between 76° and 78° maximizes the magnetic anisotropy, as it minimizes the energy gap between the $d_{xy}$ and $d_{x^{2}-y^{2}}$ orbitals, what in turn maximizes the splitting due to spin–orbit coupling. The larger this splitting, the larger is the magnetic anisotropy [23]. In the quantification of the magnetic anisotropy in tetrahedral cobalt complexes on the basis of experimental charge density investigations, Overgaard et al. found an energy barrier of *U*_eff_ of 342 cm^−1^ for the idealized case of $d_{xy}$ and $d_{x^{2}-y^{2}}$ orbitals being degenerated [24]. In this work, we investigate two mononuclear cobalt(II) complexes chelated by two diimidosulfinate ligands that display pronounced single‐ion magnetic anisotropy, the previously reported compound [Co{(N^*t*^Bu)_2_SPh}_2_] [23] (**1_Co**) and the newly synthesized analog [Co{(NSiMe_3_)_2_SPh}_2_] (**2_Co**). These slightly different complexes were subjected to charge density investigations based on high‐resolution X‐ray diffraction (XRD) data to obtain detailed insights into their electronic structures. This should give insight to how a subtle ligand variation effects their magnetic properties. Because both complexes adopt very similar coordination geometries at the cobalt atom, differences in *d*‐orbital populations can be correlated with changes in the ligand environment. In addition, the reliability of the chosen methods can be benchmarked by trying to describe so subtle differences. With this in mind, complementary quantum chemical calculations were performed to provide orbital population data to compare to experimental findings. To further clarify the ligand–metal interactions, the structures of the free protonated ligands were compared with those in the cobalt coordination sphere. Finally, magnetic characterizations were carried out to evaluate and compare the magnetic anisotropy and relaxation dynamics of **1_Co** and **2_Co**.

## 2 Results and Discussion

**FIGURE 1:**
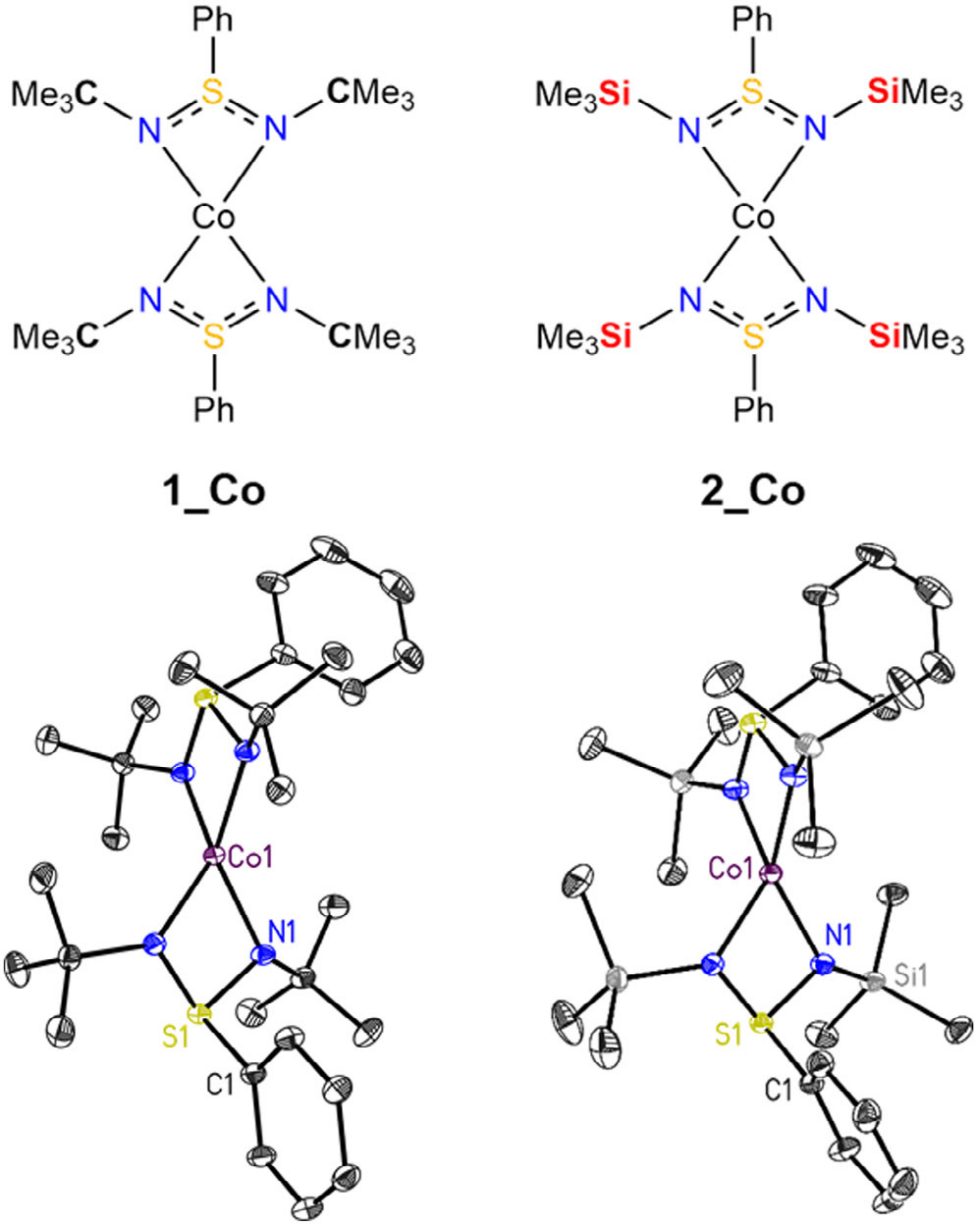
Lewis diagrams and anisotropic displacement parameters of **1_Co** and **2_Co**.

The cobalt(II) complex [Co{(NSiMe_3_)_2_SPh}_2_] (**2_Co**) was synthesized through the salt metathesis reaction of the lithium diimidosulfinate ([Li{(NSiMe_3_)_2_SPh}]_2_·O^*n*^Bu_2_, **2_Li**) with anhydrous cobalt dichloride in tetrahydrofuran under inert atmosphere. Purple block‐shaped crystals suitable for XRD analysis were obtained from the saturated product solution in a mixture of *n*‐hexane and tetrahydrofuran at −36°C with 85% yield. The cobalt in **2_Co** is chelated by two diimidosulfinate ligands with cobalt in the oxidation state +II (Figure 1).

### 2.1 Structure

To obtain initial insights into the influence of a slight change in ligand substitution, the geometries of both complexes **(1_Co** and **2_Co**) were compared, along with the geometries of the protonated metal‐free ligands (**1_H** and **2_H**). The crystal packing of **1_H** and **2_H** is dominated by hydrogen bonds. To avoid influences of that on the structure, the geometries were optimized.

**FIGURE 2:**
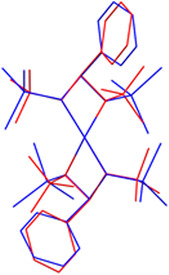
Superposition plot of **1_Co** (blue) and **2_Co** (red).

The geometries of both complexes (**1_Co** and **2_Co**) are very similar (see Figure 2). The most important bond lengths and angles are shown in Table 1. The major difference is that all relevant bond angles of **2_Co** are slightly larger compared to **1_Co**. This is attributed to the larger steric demand of the tms groups compared to the ^*t*^Bu groups. The larger N–Co–N bite angle is the most important angle when it comes to the discussion of the magnetic properties. The closer the bite angle is to 76°–78°, the better are the magnetic properties [23]. The Si—N bond is longer than the C—N bond, as expected. The S—N bonds are slightly shorter in **2_Co**, whereas the Co—N bonds are slightly longer compared to the Co—N bond lengths of **1_Co**. These differences arise from the more pronounced electron‐releasing character of the tms substituent. According to Overgaard et al. [24] and in agreement to ligand field theory, the interaction between ligand and metal through the $d_{xz}$ and $d_{yz}$ orbitals is antibonding. Therefore, an increase in ligand‐to‐metal charge transfer should weaken the covalent part of the bond, what is consistent with the longer ligand–metal bond distances in **2_Co**.

**TABLE 1:** Selected bond distances [Å] and angles [°] for **1_H**, **1_Co**, **2_H**, and **2_Co**.

Parameter [Å]/[°]	**1_H** a	1_Co	**2_H** b	2_Co
Co–N		2.00841(19)−2.01398(19)		2.0195(2)−2.0283(2)
S–N	1.5377	1.6332(2)−1.6366(2)	1.5197	1.6121(3)−1.6234(3)
S–NH	1.6589		1.6497	
S–C	1.7960	1.8031(3)−1.8048(3)	1.7929	1.7970(3)−1.7985(3)
C/Si–N	1.4719	1.4769(3)−1.4786(3)	1.7007	1.7317(8)−1.7376(8)
C/Si–NH	1.4777		1.7522	
N–Co–N		72.742(9)−72.767(9)		73.329(11)−73.635(10)
N–S–N	110.25	93.744(11)−93.745(11)	109.97	96.594(13)−97.252(13)
S–N–C/Si	120.24–122.84	118.447(15)−119.718(15)	126.39–131.5	122.312(15)−125.253(15)

a Geometry optimized structure of IQUHOU from the CCDC [25].

b Geometry optimized structure of WECWIK from the CCDC [26].

Comparing the bond lengths of the cobalt complexes to the protonated ligands [25, 26], it is obvious that the S—N bond lengths of the cobalt complex are merged compared to the clearly different distances in the latter. The N–S–N and S–N–C/Si angles are decreasing upon coordination. The differences between the protonated ligand **1_H** compared with the corresponding ligand of **1_Co** are more pronounced than those observed between **2_H** and the ligand in **2_Co**, indicating that the ligand core in **1_Co** experiences slightly greater strain.

### 2.2 Magnetic Properties

**FIGURE 3:**
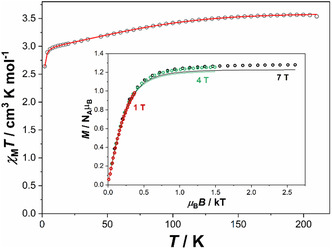
Temperature dependency of **2_Co** of the magnetic susceptibility and temperature (*χ*_M_ *T*) from 210 K to 2 K with an applied dc field of *H*_dc_ = 5000 Oe, with *χ*_M_ *T* = 3.66 cm^3^mol^−1^K at 200 K. The VTVH magnetization measurements (inset) are presented as *M* versus *µ*_*B*_ *B* at 7 T, 4 T, and 1 T for **2_Co** at 2 K. The solid lines represent the calculated curve fits. VTVH = Variable‐temperature/variable‐field.

Magnetic characterization of **2_Co** was performed using a polycrystalline powdered sample coated with a low‐viscosity inert oil to prevent any torque effects in the magnetic field. At 200 K, the *χ*_M_ *T* value of 3.66 cm^3^mol^−1^K is significantly higher than the spin‐only value expected for an isolated cobalt(II) ion (*S* = 3/2, *g* = 2.0, *χ*_M_ *T*  =  1.875 cm^3^mol^−1^K; Figure 3). This increase reflects a significant orbital contribution to the magnetic moment. Upon cooling, the *χ*_M_ *T* value gradually decreases to 6 K and then drops steeply, reaching 2.64 cm^3^mol^−1^K at 2 K. Since intermolecular interactions between the Co(II) atoms are most likely negligible due to the relatively large separation of 9.659(2) Å, the pronounced decrease observed below 6 K is best attributed to significant magnetic anisotropy. Variable‐temperature/variable‐field (VTVH) magnetization and dc susceptibility data were fitted assuming g_*x*_ = g_*y*_ < g*_z_* yielding g_*x*_ = g_*y*_ = 2.32, g*_z_* = 3.24, and *D*_exp_ = −138.9 cm^−1^ (Figure 3, inset). This axial zero‐field splitting parameter (*D*_exp_) is more negative than those reported for related cobalt diimidosulfinate complexes (*D*_exp_ = −75.5 to −114 cm^−1^) [23]. The larger negative *D* value of **2_Co** correlates with its wider N–Co–N bite angle of 73.329(11)° and 73.635(10)°, which approaches the idealized angle of 77.4^°^ predicted for an *S* = 3/2, *L* = 2 system. This wider N–Co–N bite angle appears to favor the ligand field stabilization with a theoretical energy gap (*U*) of 277.9 cm^−1^ (|2*D*|) between the ground state *M*_S_ = ±3/2 Kramers doublet (KD) [27] and the excited state *M*_S_ = ±1/2 KD and thereby enhances magnetic anisotropy in comparison to previously reported analogs.

**FIGURE 4:**
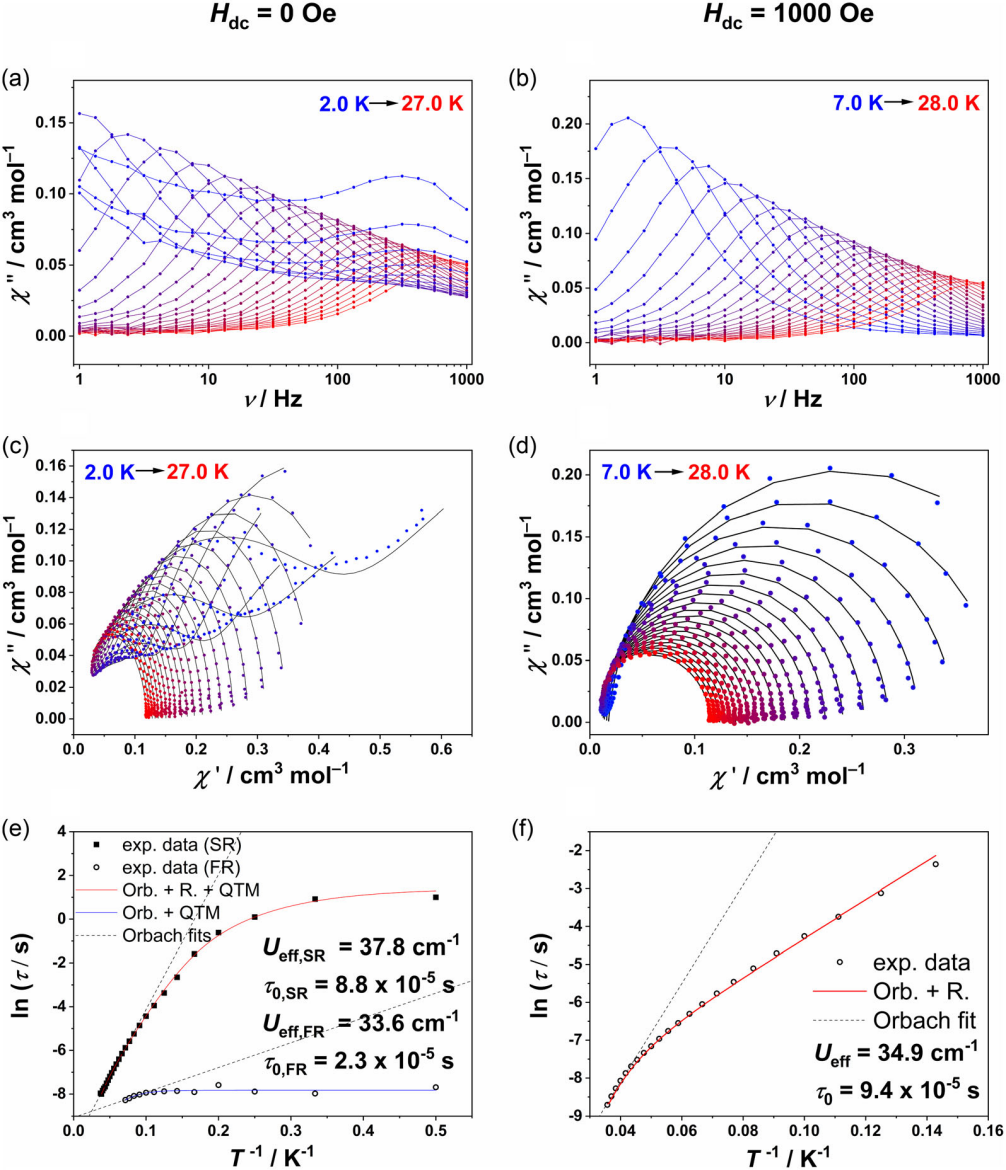
Out‐of‐phase (*χ*_M_”) ac susceptibility for **2_Co** at zero dc field (a) and 1000 Oe dc field (b). Cole–Cole plots showing slow (SR) and fast (FR) relaxation under zero field (c) and a single process after QTM suppression at 1000 Oe (d). Arrhenius plots with linear Orbach fit (dotted line) and combined fits including Orbach (Orb.), Raman (R.), and QTM relaxation processes (solid line) under zero field (e) and 1000 Oe (f). QTM = Quantum tunneling mechanism of magnetization.

To investigate the magnetic relaxation dynamics, alternating current (ac) susceptibility measurements were conducted for complex **2_Co** in the frequency range from 0.1 to 1000 Hz under zero dc field (Figure 4a). Frequency‐dependent maxima in the out‐of‐phase susceptibility (*χ*_M_”) were observed between 2 and 27 K. At lower temperatures (2–14 K), an additional maximum with weaker frequency dependence was detected, corresponding to a fast magnetic relaxation process (FR), whereas the slow relaxation process (SR) exhibited maxima in the range from 7 to 27 K. The weaker frequency dependence of the FR suggests a significant contribution of the quantum tunneling mechanism (QTM) to the relaxation mechanism. To confirm this further, ac susceptibility measurements were performed under an applied optimal dc field of 1000 Oe to suppress quantum tunneling effects (Figure 4b). Under these conditions, only a single relaxation process was observed between 7 and 28 K, demonstrating that QTM plays a dominant role in the FR observed at zero field. The in‐phase (*χ*_M_’) and out‐of‐phase (*χ*_M_”) susceptibilities were combined into Cole–Cole plots for both zero and 1000 Oe dc fields and fitted using a generalized Debye model (Figure 4c,d). The extracted temperature‐dependent relaxation times were fitted considering Orbach (Orb.), Raman (R.), and quantum tunneling relaxation mechanisms according to Equation (1).

(1) $$\tau^{-1}=\tau_{0}^{-1}e^{-U_{\mathrm{eff}}/k_{B}T}+CT^{n}+\tau_{\mathrm{QTM}}^{-1}$$

### 2.3 Orbach Raman Quantum Tunneling Mechanism

To minimize overparameterization, the fitting employed the smallest possible number of parameters. The resulting parameters are shown in Table 2 (additional details are provided in Supporting Information (SI) Table S1). The fast relaxation process (FR) of complex **2_Co** at zero field was satisfactorily fitted using Orbach and QTM mechanisms, showing good agreement with experimental data despite notable scattering between 2 and 6 K. The slow relaxation process (SR) was well described by incorporating all three magnetic relaxation mechanisms. Under an applied dc field of 1000 Oe, the relaxation behavior of **2_Co** was adequately modeled using Orbach and Raman processes.

**TABLE 2:** Selected magnetic data for **1_Co** and **2_Co**.

Compound, process	1_Co	1_Co	**2_Co**, FR^a^	**2_Co**, SR^b^	2_Co
*H*_dc_/Oe	0	1500	0	0	1000
*T*_AC_ c/K	2–20	6.5–22	2–14	2–27	7–28
*τ*_0_/10^−6^ s	35(12)	51(6)	23(30)	80(10)	94(11)
*U*_eff_/cm^−1^	36(3)	35.6(6)	34(13)	37.8(1.1)	34.9(8)
C/K^‐n^s^−1^	6.8(6.9) · 10^4^	6.0(8.7) · 10^−7^	—	22(8) · 10^−4^	3.6(4.4) · 10^−7^
*n*	4.9(4)	7.2(5)	—	4.11(12)	6.9(4)
*τ*_QTM_/s	0.02	—	40.0(1.7) · 10^−5^	4(2)	—

a Fast relaxation process.

b Slow relaxation process.

c Minimum and maximum temperature at which a maximum is observed in AC current measurement.

For comparison, the raw data of the previously reported compound **1_Co** were fitted including the QTM process (Figure S5 c in ESI† (Electronic Supporting Information)), showing good agreement with the experimental data. All relaxation mechanisms exhibited comparable energy barriers (34–37.8 cm^−1^) and relaxation times (2.3–9*.*4·10^−5^ s). The more negative axial zero‐field splitting parameter (*D*_exp_(**1_Co**) = −114 cm^−1^; *D*_exp_(**2_Co**) = −139 cm^−1^) and the observation of maxima in the out‐of phase susceptibility at higher temperatures indicate that compound **2_Co** exhibits slightly better single molecule magnet performance compared to **1_Co**, although both compounds remain largely *on par*. Temperature‐dependent hysteresis measurements of **2_Co** performed at a sweep rate of 50 mT/s revealing a butterfly shaped open hysteresis loop extending up to 3.8 K (Figure S4 in ESI†). The collapse of the hysteresis loop near zero field, which leads to the characteristic butterfly shape, reflects QTM, a phenomenon frequently observed in previously reported cobalt(II) SMMs [22, 28, 29, 30, 31, 32].

### 2.4 Structure and Charge Density

To gain further insight in the ligand–metal interaction and the *d*‐orbital populations, high‐resolution XRD datasets were collected for both compounds, **1_Co** and **2_Co**. Multipole refinements according to the formalism by Hansen and Coppens [33] yielded charge density distributions of good quality, which were subject of deeper analysis. This was done using Bader's quantum theory of atoms in molecules (QTAIM) [34], in which the electron density and its curvature at the bond critical point (BCP) are analyzed (Table 3), as well as the Bader charges (Table 4) of the atoms.

**TABLE 3:** Selected charge density properties of **1_Co** and **2_Co** at the BCP.

		*ρ*_(BCP)_, eÅ^–3^		∇^2^ *ρ*_(BCP)_, eÅ^–5^		*ε*_(BCP)_		|*V*|/*G*	
		1_Co	2_Co	1_Co	2_Co	1_Co	2_Co	1_Co	2_Co
Exp.	C/Si–N	1.76	0.98	−13.64	4.80	0.04	0.01	2.67	1.66
	S–N	1.69	1.72	−9.17	−9.09	0.15	0.10	2.43	2.41
	Co–N	0.61	0.57	9.17	8.70	0.14	0.07	1.18	1.15
	C–S	1.32	1.30	−5.91	−5.37	0.04	0.02	2.43	2.38
Theo^a^	C/Si–N	1.74	0.95	−15.88	6.44	0.04	0.03	2.86	1.57
	S–N	1.79	1.81	−13.23	−12.82	0.16	0.12	2.61	2.71
	Co–N	0.46	0.44	7.21	9.37	0.03	0.03	1.09	1.06
	C–S	1.36	1.39	−6.99	−7.44	0.03	0.03	2.48	2.50

a Theoretical structure factors were calculated with the method from Genoni [35] in a 20x20x20 Å box.

**TABLE 4:** Bader charges of **1_Co** and **2_Co**.

Atom	1_Co	2_Co
Co	0.92	0.86
N	−1.14	−1.45
S	0.97	0.96
C/Si	0.49	2.46
CMe_3_/SiMe_3_	0.51	0.94
Ph	−0.14	−0.33

**FIGURE 5:**
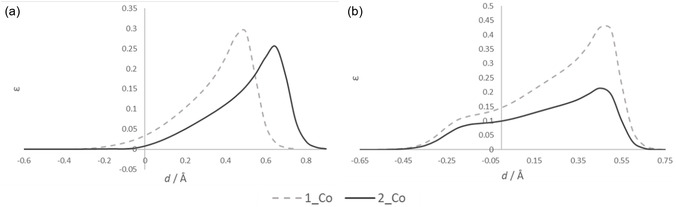
The ellipticity along (a) the C/Si—N bond and (b) the S—N bond.

The differences between the C—N and the Si—N bonds in the cobalt complexes are in good agreement with literature reports [36]. The C—N bonds exhibit covalent character, as evidenced by a high electron density, a high negative Laplacian, and a ratio of potential to kinetic energy [37, 38] greater than two at the BCP. The Si—N bonds exhibit an intermediate bond character, with a lower electron density at the BCP, a small positive Laplacian, and a |*V*|/*G* ratio between one and two (Table 3). The bond characters are also well mirrored in the valence shell charge concentrations (VSCCs) with accumulations in the C–N binding region, and the typical shape of the Laplacian for the Si—N bond, where the charge accumulation is very much distorted toward the nitrogen atom, while the silicon has an depletion of electron density [39]. The ellipticity is not evenly distributed along the C/Si—N bond, but most pronounced at the most electronegative nitrogen atom, as anticipated for polar bonds (Figure 5). The slight difference in the ellipticity between **1_Co** and **2_Co** predominantly arises from variations in bond polarizations rather than from inherent *π* character [40].

**FIGURE 6:**
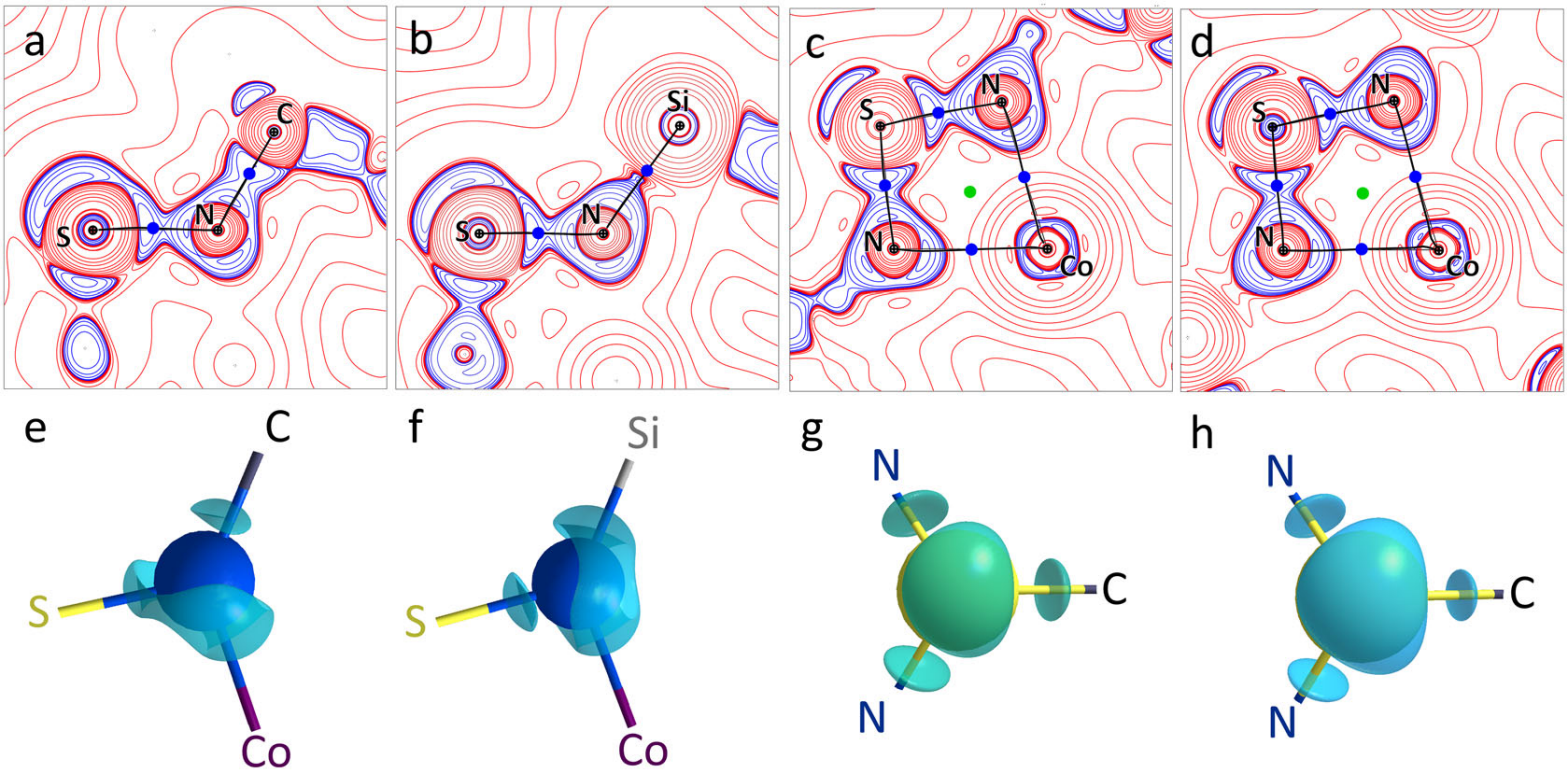
The VSCCs of **1_Co** (a,c,e, and g) and **2_Co** (b,d,f, and h) of the S–N–C/Si plane (a,b), the N–S–N plane (c,d) and 3D cubes around a nitrogen atom (e,f, isolevel: 38 eÅ^−5^) and sulfur (g,h, isolevel: 10 eÅ^−5^). VSCCs = Valence shell charge concentrations.

In examining the influence of this to the remaining bonds, only insignificant changes were observed. The VSCCs are looking the same for both compounds along the S—N bond and along the Co—N bond, where the accumulated electron density of the nitrogen atoms interacts with the depleted regions of the cobalt atom. A slight tendency toward increased ionic character of the bonds in **2_Co** can be noted. This effect may be rationalized by an enhanced charge separation, which would increase with the electron donating capability of the Me_3_Si substituent. In addition, the bond ellipticity is reduced in **2_Co** compared to **1_Co**. The smaller influence of the nitrogen lone pair on the S—N bond of **2_Co** is also visible when looking at the VSCC around nitrogen (Figure 6). To substantiate this interpretation and to gain further insight into charge transfer within the ligands and toward the metal atom, a comparison of the corresponding Bader charges was performed (Table 4).

The most pronounced charge redistribution is observed at the nitrogen atom, where the influence of the Me_3_Si substituent is strongest. In **2_Co**, the cobalt atom itself carries a lower positive charge, indicating an increased electron donation of the ligand compared to **1_Co**. The influence of the Me_3_Si substituent on the central sulfur atom is insignificant. Consequently, the charge differences within the nitrogen–sulfur interaction in **2_Co** are more pronounced, which accounts for the slightly enhanced ionic bond character. This increase in ionic contribution stabilizes the S—N bonds, leading to their shortening. Those findings are mirrored both in the geometric features and in the properties at the BCPs.

On this basis, we embarked on the analysis of the cobalt *d*‐orbital populations in order to evaluate whether or not changes in the ligand's periphery influence these populations and, consequently, the magnetic properties.

The determination of *d*‐orbital populations relies on the assumption that there is no overlap between the metal electron density and the ligand's density [41]. As judged from the |*V*|*/G* criterion, the Co—N bond is not purely ionic. Accordingly, part of the charge transfer might not have been adequately modeled by the cobalt multipoles and has been “mopped‐up” by the nitrogen atom's multipoles instead. Therefore, the following discussion of charge transfer should be followed with some caution [42].

To get quantitative insights into the ligand‐to‐metal charge transfer, the *d*‐orbital populations are compared to results obtained from CAS‐SCF calculations. In the calculations, charge transfer between metal and ligands is definitely not included. Hence, a comparison to the experiment, which may include charge transfer, with the computational data allows to assess the impact of charge transfer on the *d*‐orbital populations.

**FIGURE 7:**
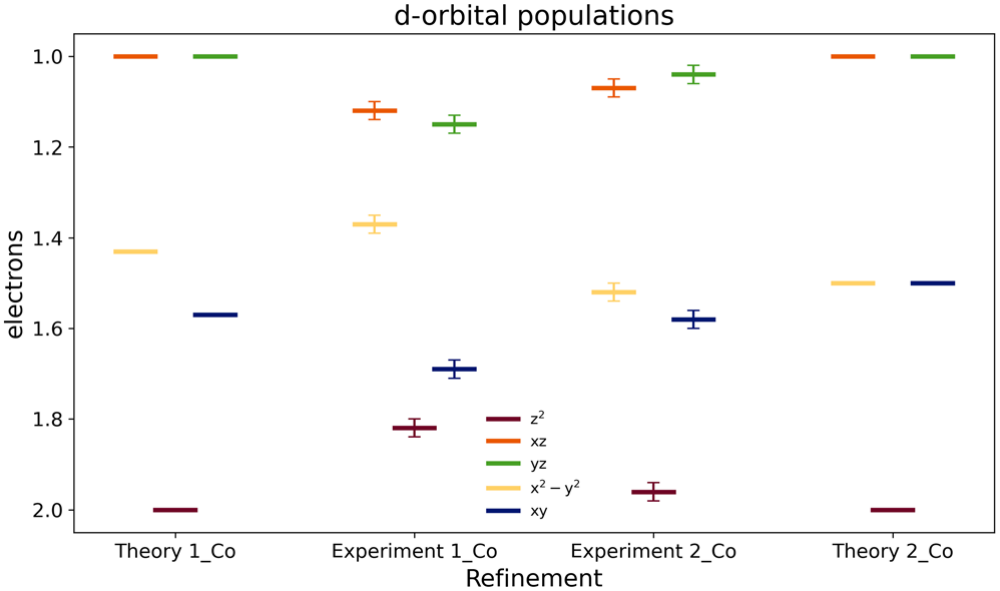
The *d*‐orbital populations from experiment and directly from the theory (without multipole refinement). The *x*‐axis has the lowest value at the top, to be more intuitive as the orbitals with the lowest occupancy are the ones with highest energy. Experimental errors were determined by xdprop [43].

The energetical order is in good agreement between experiment and theory (Figure 7, Table 5). As predicted by ligand field theory, in both complexes, the $d_{xz}$ and $d_{yz}$ orbitals are almost degenerated (red and green in Figure 7). Their occupations are slightly higher than predicted by the calculations. According to Overgaard et al. [24], these orbitals exhibit antibonding interactions with the ligands. Since the increase in electron density is somewhat greater in **1_Co**, stronger orbital–ligand interaction appears to occur in this complex. However, this contradicts the initial assumption that **2_Co** should exhibit stronger interactions due to the stronger electron‐donating character of the Me_3_Si group compared to the ^*t*^Bu group, and it also conflicts with the Bader charges at cobalt. Moreover, as the interaction is supposed to be antibonding, longer Co—N bonds in **1_Co** are anticipated. Experimentally, however, the bond lengths are slightly longer in **2_Co**.

**TABLE 5:** *d*‐orbital populations from the multipole refinement and theoretical calculations (without multipole refinement).

Orbital	1_Co exp.	1_Co theo.	2_Co exp.	2_Co theo.
*z* ^2^	1.82 (25.5 %)	2 (28.57 %)	1.96 (27.3 %)	2 (28.57 %)
*xz*	1.12 (15.7 %)	1 (14.29 %)	1.07 (14.9 %)	1 (14.29 %)
*yz*	1.15 (16.0 %)	1 (14.29 %)	1.04 (14.5 %)	1 (14.29 %)
*x* ^2^‐*y* ^2^	1.37 (19.1 %)	1.43 (20.43 %)	1.52 (21.2 %)	1.50 (21.43 %)
*xy*	1.69 (23.7 %)	1.57 (22.43 %)	1.58 (22.1 %)	1.50 (21.43 %)
Total 3d	7.15 (100 %)	7 (100 %)	7.16 (100 %)	7 (100 %)
4s	1.33	0	1.14	0

*Note:* Errors were determined by xdprop [43].

The $d_{x^{2}-y^{2}}$ orbital is in both cases in good agreement to the calculations, while the $d_{xy}$ orbital is more occupied in both cases, again more pronounced in **1_Co**. Following the arguments of Overgaard et al. [24], this deviation could arise from orbital mixing with either the 4s orbital or the energetically proximate $d_{z^{2}}$ orbital. This orbital is in **1_Co** less populated than predicted. Thus, it seems likely that the $d_{xy}$ orbital in **1_Co** is mixing mostly with $d_{z^{2}}$, as those orbitals are close in energy. In **2_Co**, the gap between the $d_{xy}$ and the $d_{z^{2}}$ orbital is larger, leading to less mixing between those orbitals.

In summary, the substituent modification exerts only a minor influence on the cobalt atom. While subtle effects are observed in structural parameters, bond properties, and charge distribution, the *d*‐orbital populations themselves do not exhibit significant differences. In fact, the orbital population analysis conveys a picture opposite to that suggested by geometry, charge, and bond property analyses. This is an interesting finding, as the *d*‐orbital population is an important and widely used property, when it comes to SMMs.

During the optimization of the refinement process, it became evident that the *d*‐orbital populations were sensitive to the choice of the X‐ray data integration method. Therefore, a second dataset was collected (for details, see ESI†). This dataset provided higher resolution, as the crystal was considerably larger, which also resulted in improved quality indicators. However, the *d*‐orbital populations (and other related properties) proved to be even less reliable, most probably due to absorption effects. In fact, the deviations resembled those reported by Overgaard, Meyer et al. [32] The $d_{xz}$ and $d_{yz}$ orbitals were no longer degenerate and the $d_{xy}$ orbital was found to be higher populated than the $d_{z^{2}}$ orbital. For this reason, the first dataset was used for comparison. Nonetheless, also for this reason the *d*‐orbital populations should be seen with caution. To fully understand the unreliable behavior of the *d*‐metal multipoles, datasets of more complexes showing this kind of *d*‐orbital population clearly are needed.

From the *d*‐orbital populations, the splitting of the KD can be derived, which represents the theoretical barrier to spin inversion (Table 6). The theoretical value Δ_K,theo_ corresponds to the ligand‐field splitting (and thereby to the bite angle), while neglecting any contributions from charge transfer. In contrast, the experimentally derived value Δ_K,ED_ also includes the effects of charge transfer. The zero‐field splitting parameter *D*, obtained from magnetic susceptibility measurements (*D*_exp,SQUID_), provides an additional experimental measure of the anisotropy barrier under experimental conditions.

**TABLE 6:** Energy difference between the two KDs of **1_Co** and **2_Co**.

Method	1_Co	2_Co
Δ_K, theo_ /2	−155	−170
Δ_K, ED_ /2	−134	−163
*D*_theo_	−148	−150
*D*_exp, SQUID_	−114 [23]	−139

The barrier is higher for **2_Co**, which is consistent with expectations based on its slightly larger bite angle. As anticipated, the value decreases when charge transfer processes are considered, as the degeneracy of the $d_{xy}$ and $d_{x^{2}-y^{2}}$ orbital is lifted. The spread between the experimental values is large, comparable to the magnitude of the difference between the two compounds. Therefore, it is important to always use the same method when comparing different complexes. So, all in all it seems as every method by itself is able to represent the change in magnetic properties properly, but the absolute values are not reliable yet. This is also due to the fact that the structure and charge density are not the only properties that are relevant for the magnetic properties, but also effects like spin–phonon coupling [44, 45, 46].

## 3 Conclusion

The cobalt(II) complex [Co{(NSiMe_3_)_2_SPh}_2_] (**2_Co**) was successfully synthesized and studied in direct comparison with the known analog [Co(N^t^Bu)_2_SPh_2_] (**1_Co**). Both compounds show very similar cobalt coordination geometries, with only minor variations arising from the different substituents. High‐resolution XRD experiments combined with multipole refinement and QTAIM analysis point to slightly altered bonding characteristics in **2_Co**, in particular a somewhat stronger ionic contribution and a marginally higher electron density at the cobalt atom. The trend to stronger ionic bonds is in line with the Bader charge analysis. The interpretation of the experimental *d*‐orbital populations with respect to charge transfer processes proved less straightforward, as they partly deviate from findings from other properties and differ in addition between different single crystal diffraction datasets. However, the $d_{\mathrm{xy}}$ and $d_{x^{2}-y^{2}}$ orbitals, which are of interest for the magnetic properties, are not affected by ligand‐to‐metal charge transfer. Therefore, the Δ_K,ED_ value should be reliable.

Magnetically, complex **2_Co** displays a large axial anisotropy (*D* = –138.9 cm^−1^) and field‐dependent relaxation processes in the ac susceptibility. The data suggest that QTM plays a role at low temperatures, while under applied dc fields relaxation becomes dominated by thermally activated processes. Compared with **1_Co**, the overall magnetic behavior is largely similar, but **2_Co** shows indications of slightly enhanced SMM features. Benchmarking, however, of experimental and computational approaches seem not to be valid yet. Clearly more data are needed to pin down the various contributions.

## 4 Experimental Section

### 4.1 General Procedure

All reactions were performed in oven‐dried glassware under dry argon, using Schlenk techniques or an argon glove box. All solvents were distilled from sodium or potassium and stored over 3 Å molecular sieve prior to use.

**2_Li**: [Li{(NSiMe_3_)_2_SPh}]_2_·O*^n^* Bu_2_ was synthesized according to the procedure of Stalke et al. [47] with phenyl lithium in di‐*n*‐butyl ether instead of phenyl lithium in diethyl ether, resulting in a crystal structure where the diethyl ether is exchanged with di‐*n*‐butyl ether (see ESI† crystal data). The cobalt dichloride was purchased from Sigma‐Aldrich at a 99.999% purity level and used as received. The ^1^H‐ and ^13^C‐NMR spectroscopy was performed on a Bruker Avance Neo 400 MHz spectrometer at 298 K and referenced to the deuterated solvent signal (THF‐d8). The ESI/mass spectrometry (MS) data were recorded on a Bruker HCT Ultra Spectrometer. The elemental analysis measurements of the mass fractions of carbon, hydrogen, nitrogen, and sulfur were performed by the microanalytical laboratory of the Institute of Inorganic Chemistry at the University of Göttingen using an Elementar Vario EL3 analyzer.

**2_Co**: [Li{(NSiMe_3_)_2_SPh}]_2_·O^*n*^Bu_2_ (273.9 mg, 0.39 mmol, 1 eq.) and CoCl_2_ (50 mg, 0.39 mmol, 1 eq.) were suspended in 3 mL tetrahydrofuran and stirred for 2 days at ambient temperature. After 30 min of stirring, the formed solution underwent a color change from blue to purple. The reaction mixture was then concentrated in vacuo, and 2 mL *n*‐hexane were added. The precipitating lithium chloride was centrifuged off, and crystals suitable for XRD analysis were obtained from a saturated solution of **2_Co** in a mixture of *n*‐hexane and tetrahydrofuran at −36^°^C. Crystalline yield: 85%. ^1^H NMR (400.3 MHz, THF d_8_, ppm) δ: 52.02 (br, W_1*/*2_ = 1089 Hz, 4H, *ortho*‐C_6_H_5_), 29.12 (br, W_1*/*2_ = 86 Hz, 4H, *meta*‐C_6_H_5_), 11.31 (br, W_1*/*2_ = 37 Hz, 2H, *para*‐C_6_H_5_), −29.84 (br, W_1*/*2_ = 500 Hz, 18H, Si(CH_3_)_3_), −47.21 (br, W_1*/*2_ = 482 Hz, 18H, Si(CH_3_)_3_). ^13^C NMR (125.8 MHz, THF‐d_8_, ppm) δ: 274.7 (br, W_1*/*2_ = 55 Hz, 4C, *meta*‐C_6_H_5_), 261.5 (br, W_1*/*2_ = 298 Hz, *ipso*‐C_6_H_5_ or *ortho*‐C_6_H_5_), 255.9 (br, W_1*/*2_ = 295 Hz, *ipso‐*C_6_H_5_ or *ortho*‐C_6_H_5_), 213.7 (br, 2C, *para*‐C_6_H_5_), 153.2 (br, 3C, Si(CH_3_)_3_), 152.5 (br, 3C, Si(CH_3_)_3_). ESI/MS: (THF, *m/z*): 676.1 [M + C_4_H_3_]^−^. Anal. Found (Calc.) for C_24_H_46_N_4_S_2_Si_4_Co (*M* = 626.05 g/mol): C: 45.86 (46.04); H: 7.4 (7.41); N: 8.96 (8.95); S: 9.82 (10.24). Temperature‐dependent magnetic susceptibility measurements were carried out using a Quantum Design MPMS 3 with a 7 T magnet.

### 4.2 High‐Resolution X‐Ray Diffraction

Crystals of **1_Co** and **2_Co** were measured up to a resolution of 0.48 Å using for **1_Co** a diffractometer equipped with a Bruker LRA Mo rotating anode, a Bruker D8 three‐circle goniometer, SMART APEX II CCD detector, and Montel mirror optics. For **2_Co,** a diffractometer equipped with Mo IµS microfocus source, Bruker D8 four‐circle goniometer, and CMOS‐PHOTONIII detector was used. Data were integrated with SAINT [48] from the APEX4 suite [49], absorption correction was conducted using the model for strong absorbers in SADABS [50], and the error estimation was done in SADABS [50] using error model 5, which refines all *K* and an overall g. The structure was solved using SHELXT [51], and the IAM refinement was done with SHELXL [52] in the SHELXle suite [53]. For the MM refinement according to the formalism by Hansen and Coppens [33], the XD program [43] was used. The refinement was conducted stepwise by introducing one parameter after the other, and refining *κ*’ and the angles of the C—H bonds separately. Gram–Charlier parameters [54] of third order were refined for chosen carbon atoms, fulfilling Kuh's rule. Chemical constraints for all ^*t*^Bu or tms groups, respectively, were introduced, as well as for the phenyl groups, the nitrogen atoms and the sulfur atoms. Local symmetries were assumed for the atoms of the phenyl group (*C*_2v_), the tms/^*t*^Bu group (*C*_3v_), and for the sulfur atoms (C_s_). All hydrogen atoms were refined up to the quadrupole level with cylindrical symmetry. For cobalt, the second monopole was refined and interpreted as 4s‐orbital population, and only multipoles with inversion symmetry were refined. The Poincaré–Hopf rule is fulfilled, and the rfree test [55] showed no overfitting. An overall steps merged XD masterfiles as well as the input and result files are provided in the SI.

### 4.3 Calculations

The CASSCF/NEVPT2 calculations were done using the ORCA program package [56]. For input files, see SI. The geometries were optimized.

### 4.4 Refinement against Theoretical Structure Factors

The theoretical structures were calculated with the method from Genoni [35] in a 20 × 20 × 20 Å cube, and refined in the same way as the experimental data, but without refinement of scaling parameters, displacements and positions, and with refinement of *κ* parameters for the inner shell electrons.

## Supporting Information

Additional supporting information can be found online in the Supporting Information section.

## Author Contributions

**Katharina Rachuy**: writing – original draft, writing – review & editing, visualization, formal analysis, investigation, multipole refinement and Bader analyses, theoretical calculations. **Paula Stark**: writing – original draft, writing – review & editing, visualization, formal analysis, investigation, magnetic data analysis, synthesis and characterization. **Regine Herbst‐Irmer**: writing – review & editing, validation. **Dietmar Stalke**: writing – review & editing, supervision, conceptualization, funding acquisition.

## Funding

This work was supported by the Deutsche Forschungsgemeinschaft (389479699/GRK2455, 405832858).

## Conflicts of Interest

The authors declare no conflicts of interest.

## Supporting information

Supplementary Material

## Data Availability

The data that support the findings of this study are available from the corresponding author upon reasonable request.
