# Supplementary material for: Charge Density and Magnetic Properties in Cobalt(II) Single‐Molecule Magnets: Impact of Ligand Substitution
Source: Chemphyschem. 2026 May 11;27(9):e70404. doi: 10.1002/cphc.70404 (PMC13160253; doi:10.1002/cphc.70404)
Supplement: Supplementary file 1 — Supplementary Material [file CPHC-27-e70404-s001.pdf]

Charge-Density and Magnetic Properties in Cobalt(II) Single-Molecule Magnets: Impact of Ligand Substitution

Katharina Rachuy,+,[a] Paula Stark,+,[a] Regine Herbst-Irmer,[a] Dietmar Stalke*[a]

Inhalt

NMR spectroscopy.......2
Magnetic data.......3
Crystal data.......9
High resolution XRD and multipole modelling.......14
Theoretical calculations.......26
Multipole refinement against theoretical structure factors.......32

NMR spectroscopy

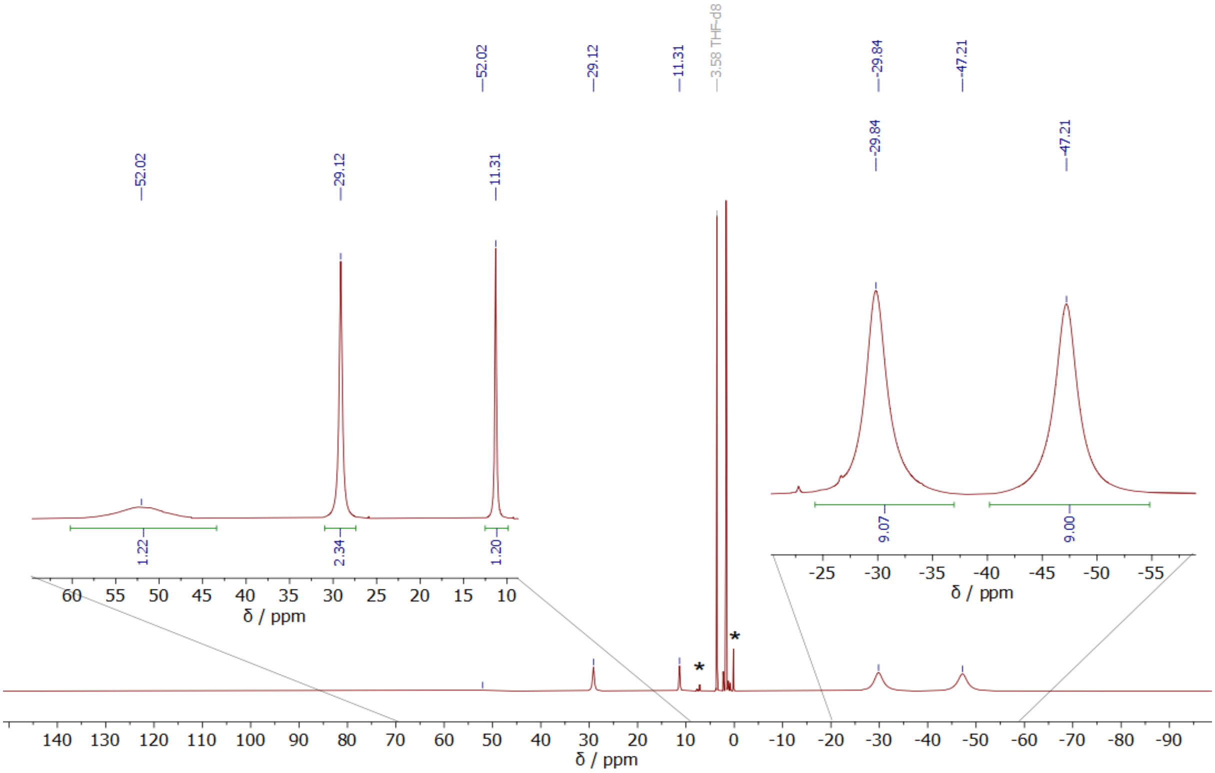

Figure S1. ^{1}H-NMR data for **2_Co** in THF-d_8 at 400.3 MHz. Signals marked with an asterisk (*) correspond to diamagnetic impurities.

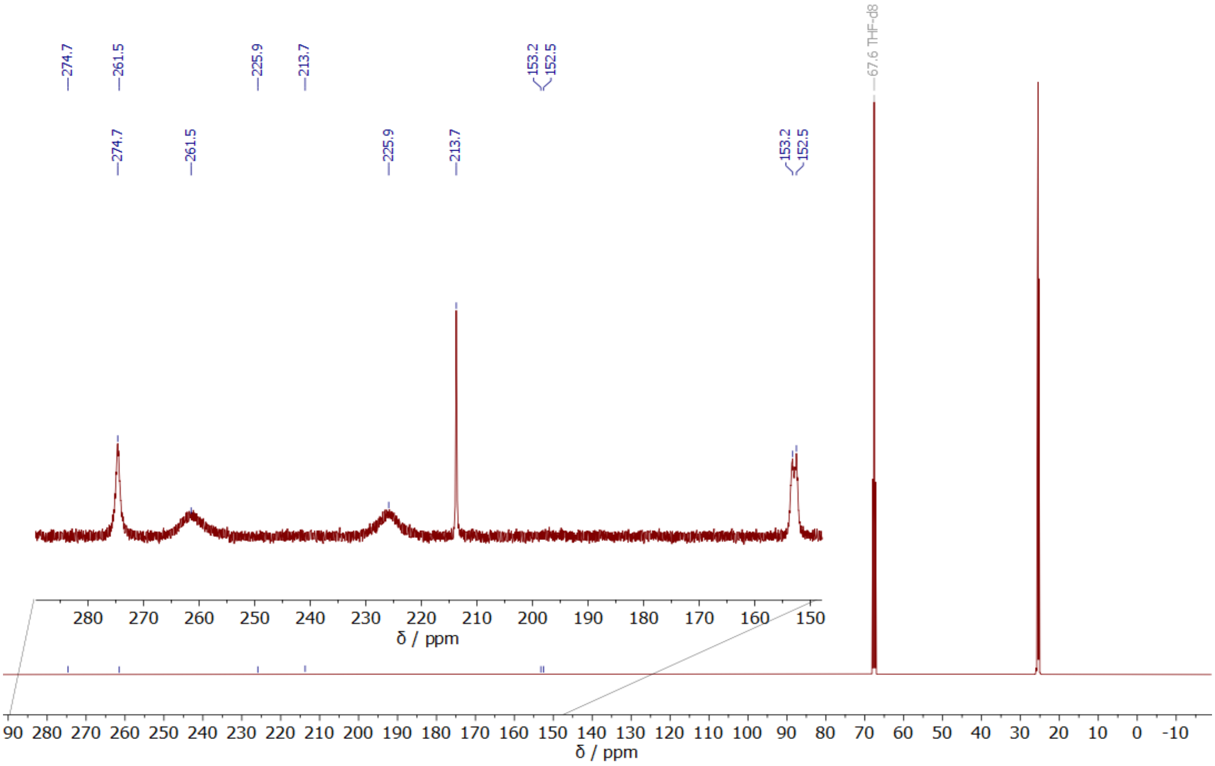

Figure S2. ^{13}C-NMR data for **2_Co** in THF-d_8 at 125.8 MHz.

Magnetic data

The dc magnetic susceptibility measurements were performed on powdered polycrystalline samples under an applied dc field of 0.5 Tesla, within a temperature range of 210.0 K to 2.0 K. The samples were covered with low-viscosity perfluoropolyether-based inert oil Fomblin Y45 to avoid torqueing and sealed in a polycarbonate capsule. The capsule was placed in a non-magnetic sample holder under an argon atmosphere. Each measured raw data point for the magnetic moment was corrected for the diamagnetic contribution of the capsule according to $\mathrm{M_{dia}} = \chi_g \cdot \mathrm{m} \cdot \mathrm{H}$, with an experimentally obtained gram susceptibility of the capsule ($\chi_g(\mathrm{capsule}) = -5.76 \cdot 10^{-7} \frac{\mathrm{emu}}{\mathrm{g \cdot Oe}}$) including the inert oil ($\chi_g(\mathrm{oil}) = -3.82 \cdot 10^{-7} \frac{\mathrm{emu}}{\mathrm{g \cdot Oe}}$). The molar susceptibility data were corrected for the diamagnetic contribution of the sample according to $\chi_{\mathrm{M,dia}}(\mathrm{sample}) = -0.5 \cdot \mathrm{M} \cdot 10^{-6} \frac{\mathrm{cm^3}}{\mathrm{mol}}$. Temperature-independent paramagnetism (TIP) was included according to $\chi_{\mathrm{calc}} = \chi + \mathrm{TIP}$.[54] To fit the dc magnetic susceptibility data and VTVH data, the Jul-2s program[55], developed by E. Bill was employed. For this purpose, the spin Hamiltonian for one cobalt $S = 3/2$ ion was used, which includes the Zeeman splitting and the zero-field splitting as follows:

$$\hat{H} = \mu_{\mathrm{B}}\left(S_x g_x B_x + S_y g_y B_y + S_z g_z B_z\right) + D\left[\hat{S}_z^2 - {}^1/_3\, S(S+1)\right] + E\left(\hat{S}_x^2 - \hat{S}_y^2\right)$$

The best fit in JulX-2s was obtained with the values $g_x = g_y = 2.316$, $g_z = 3.241$, D = -138.973 cm^{-1}, E/D = 0, TIP = 488.3 · 10^{-6} cm^{3}mol^{-1}.

Fitting of the Cole-Cole plots were obtained *via* the CC-Fit program[56,57]. The extracted values of the Cole-Cole plots were further used to construct the Arrhenius plot to determine the value of U_{eff}. Temperature dependence of the obtained relaxation times for the main process (Orbach process) were analyzed according to the Arrhenius law using:

$$\tau^{-1} = \tau_0{}^{-1} e^{-U_{\mathrm{eff}}/k_{\mathrm{B}}T}$$

The full fits were performed according to the following equation[58-60]:

$$\tau^{-1} = \tau_0{}^{-1} e^{-U_{\mathrm{eff}}/k_{\mathrm{B}}T} + CT^n + \tau_{\mathrm{QTM}}^{-1}$$

taking into account Orbach, Raman processes, and QTM relaxation processes. In the applied field of 1000 Oe, the QTM term is suppressed, and only Orbach and Raman processes are considered. The raw data files were processed using OriginPro 2020[61].

The analysis of the ac susceptibility data gave the following results:

Table S1. Selected Magnetic parameters of the cobalt complexes **1_Co** and **2_Co**.

Compound, Process	1_Co	1_Co	2_Co, FP	2_Co, SP	2_Co
$T_{Hysterse}$ / K			2 - 3.8		
H_{dc}/ Oe	0	1500	0	0	1000
T_{AC} with Maxima / K	2 - 20	6.5 - 22	2 - 14	2 - 27	7 - 28
τ_{Orb} / $\cdot 10^{-5}$ s	1.82(12)	0.04(3)	11.6(9)	3.34(15)	0.17(4)
U_{Orb}/ cm^{-1}	38.0(7)	95(11)	7.9(7)	42.9(6)	90(4)
R_{Orb}	0.99476	0.9641	0.972885	0.99762	0.990598
τ_0 / $\cdot 10^{-6}$ s	35(12)	51(6)	23(30)	80(10)	94(11)
U_{eff} / cm^{-1}	36(3)	35.6(6)	34(13)	37.8(1.1)	34.9(8)
C	6.8(6.9) $\cdot 10^{-4}$	6.0(8.7) $\cdot 10^{-7}$	-	22(8) $\cdot 10^{-4}$	3.6(4.4) $\cdot 10^{-7}$
n	4.9(4)	7.2(5)	-	4.11(12)	6.9(4)
τ_{QTM} / s	0.02	-	40.0(1.7) $\cdot 10^{-5}$	4(2)	-
R	1	0.99828	0.768174	1	1
regression technique	orthogonal distance regression	orthogonal distance regression	levenberg marquardt	orthogonal distance regression	orthogonal distance regression

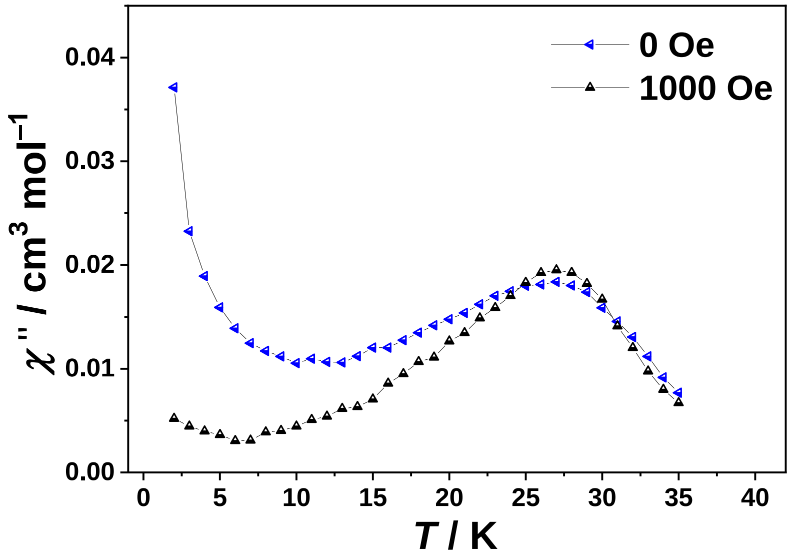

Figure S3. Temperature dependency of the imaginary part of the ac magnetic susceptibility for **2_Co** at two different dc fields: H_{dc} = 0 and H_{dc} = 1000 Oe at ν = 941.3 Hz.

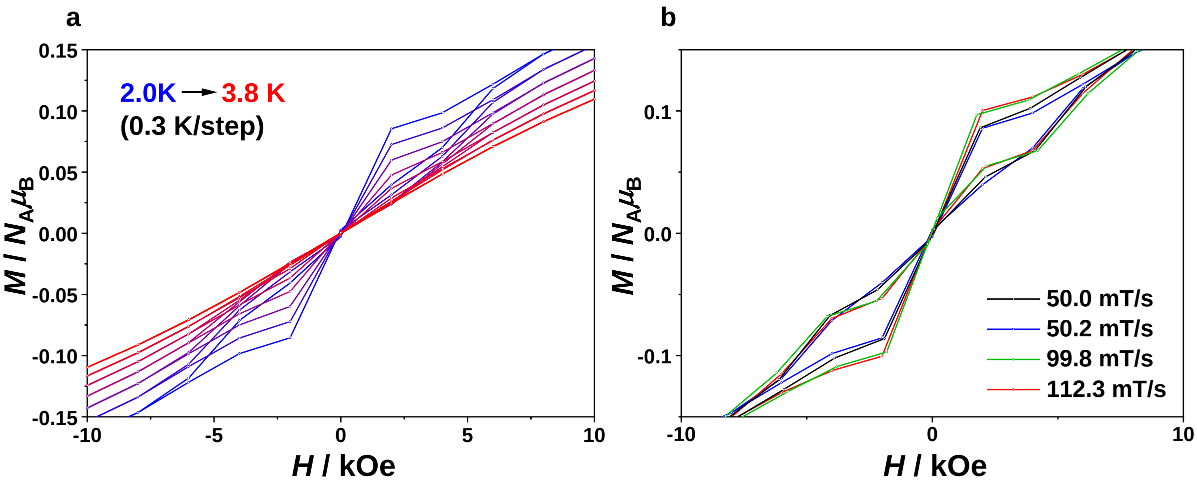

Figure S4. Magnetic hysteresis of **2_Co** at 50 mT/s sweep rate measured from 2 to 3.8 K (a) and hysteresis at 2 K with variable sweep rates under continuous (c. H_{dc}) or stabilized field (st. H_{dc}) conditions (b).

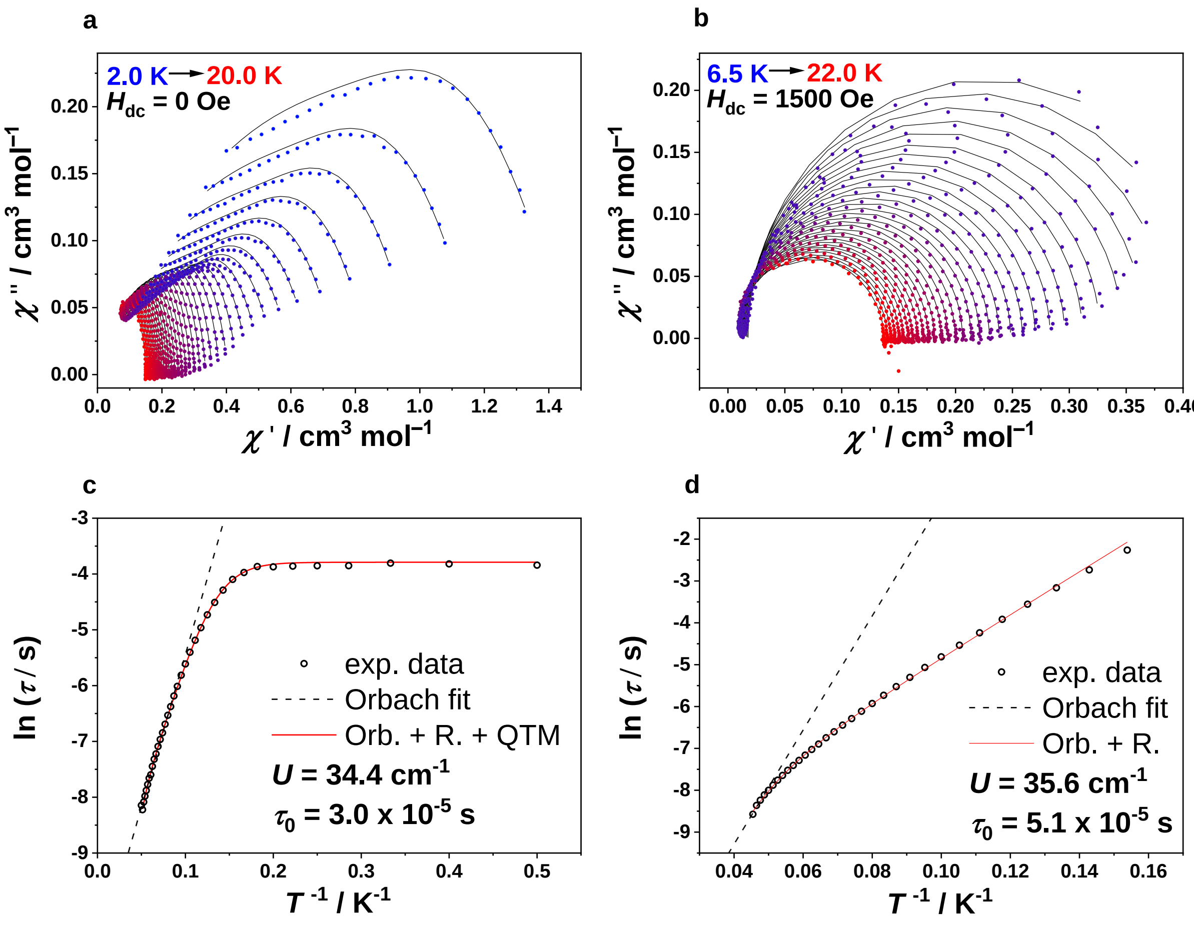

Figure S5. Cole–Cole plots (a, b) and Arrhenius plots (c, d) for **1_Co** recorded under zero dc field (a, c) and under an applied dc field of 1500 Oe (b, d). The Arrhenius plots display the global fit to all data points (red line) together with the linear fit (dotted line) to the high-temperature region, attributed to the Orbach relaxation process. Under zero field the global fit includes Orbach, Raman and quantum tunnelling of the magnetization (QTM) contributions, whereas under an applied field of 1500 Oe the QTM contribution is suppressed.

Table S2. Best fit parameters to the generalized Debye model for two processes for **2a** in zero dc field.

T / K	χ_T/ cm^{3}mol^{-1}	$\Delta\chi_{S,FP}$/ cm^{3}mol^{-1}	τ_{FP} / s	α_{FP}	$\Delta\chi_{S,SP}$ / cm^{3}mol^{-1}	τ_{SP} / s	α_{SP}	Residual
2	$3.14\cdot10^{-43}$	1.27	$1.01\cdot10^{-3}$	0.64	0.24	$2.15\cdot10^{-2}$	0.23	$1.48\cdot10^{-3}$
2.5	$2.75\cdot10^{-43}$	0.91	$5.98\cdot10^{-4}$	0.63	0.29	$2.19\cdot10^{-2}$	0.30	$5.19\cdot10^{-4}$
3	$2.41\cdot10^{-43}$	0.74	$4.89\cdot10^{-4}$	0.62	0.27	$2.22\cdot10^{-2}$	0.30	$3.15\cdot10^{-4}$
3.5	$2.35\cdot10^{-43}$	0.61	$4.01\cdot10^{-4}$	0.61	0.26	$2.12\cdot10^{-2}$	0.33	$2.17\cdot10^{-4}$
4	$2.42\cdot10^{-43}$	0.52	$3.44\cdot10^{-4}$	0.61	0.24	$2.12\cdot10^{-2}$	0.33	$1.59\cdot10^{-4}$
4.5	$2.29\cdot10^{-43}$	0.46	$3.50\cdot10^{-4}$	0.61	0.21	$2.11\cdot10^{-2}$	0.33	$1.72\cdot10^{-4}$
5	$2.21\cdot10^{-43}$	0.42	$3.41\cdot10^{-4}$	0.61	0.20	$2.08\cdot10^{-2}$	0.32	$1.50\cdot10^{-4}$
5.5	$1.92\cdot10^{-43}$	0.40	$3.83\cdot10^{-4}$	0.61	0.17	$2.09\cdot10^{-2}$	0.28	$1.62\cdot10^{-4}$
6	$2.05\cdot10^{-43}$	0.37	$3.67\cdot10^{-4}$	0.61	0.15	$1.88\cdot10^{-2}$	0.24	$1.73\cdot10^{-4}$
6.5	$3.10\cdot10^{-43}$	0.33	$3.40\cdot10^{-4}$	0.59	0.14	$1.66\cdot10^{-2}$	0.19	$2.41\cdot10^{-4}$
7	$3.14\cdot10^{-43}$	0.30	$2.82\cdot10^{-4}$	0.57	0.14	$1.37\cdot10^{-2}$	0.15	$2.81\cdot10^{-4}$
7.5	$2.70\cdot10^{-43}$	0.27	$2.31\cdot10^{-4}$	0.56	0.14	$1.10\cdot10^{-2}$	0.13	$3.20\cdot10^{-4}$
8	$6.06\cdot10^{-43}$	0.24	$1.88\cdot10^{-4}$	0.53	0.14	$8.81\cdot10^{-3}$	0.11	$3.36\cdot10^{-4}$
8.5	$3.56\cdot10^{-38}$	0.22	$1.52\cdot10^{-4}$	0.51	0.14	$6.99\cdot10^{-3}$	0.10	$3.22\cdot10^{-4}$
9	$1.04\cdot10^{-33}$	0.20	$1.30\cdot10^{-4}$	0.48	0.14	$5.59\cdot10^{-3}$	$8.59\cdot10^{-2}$	$3.59\cdot10^{-4}$
9.5	$1.06\cdot10^{-33}$	0.18	$1.09\cdot10^{-4}$	0.46	0.14	$4.51\cdot10^{-3}$	$8.27\cdot10^{-2}$	$3.48\cdot10^{-4}$
10	$1.79\cdot10^{-33}$	0.16	$9.84\cdot10^{-5}$	0.45	0.14	$3.65\cdot10^{-3}$	$7.62\cdot10^{-2}$	$2.57\cdot10^{-4}$
10.5	$1.85\cdot10^{-33}$	0.15	$8.59\cdot10^{-5}$	0.42	0.14	$2.98\cdot10^{-3}$	$6.99\cdot10^{-2}$	$2.52\cdot10^{-4}$
11	$1.36\cdot10^{-33}$	0.14	$7.34\cdot10^{-5}$	0.40	0.14	$2.44\cdot10^{-3}$	$6.76\cdot10^{-2}$	$2.20\cdot10^{-4}$
11.5	$1.86\cdot10^{-33}$	0.13	$7.08\cdot10^{-5}$	0.38	0.13	$2.06\cdot10^{-3}$	$5.97\cdot10^{-2}$	$2.27\cdot10^{-4}$
12	$2.38\cdot10^{-33}$	0.12	$5.78\cdot10^{-5}$	0.35	0.13	$1.70\cdot10^{-3}$	$6.17\cdot10^{-2}$	$1.85\cdot10^{-4}$
12.5	$5.08\cdot10^{-33}$	0.11	$5.63\cdot10^{-5}$	0.33	0.13	$1.46\cdot10^{-3}$	$4.76\cdot10^{-2}$	$1.88\cdot10^{-4}$
13	$3.83\cdot10^{-33}$	0.10	$4.97\cdot10^{-5}$	0.30	0.13	$1.24\cdot10^{-3}$	$4.60\cdot10^{-2}$	$1.59\cdot10^{-4}$
13.5	$1.37\cdot10^{-28}$	$9.70\cdot10^{-2}$	$4.63\cdot10^{-5}$	0.29	0.13	$1.06\cdot10^{-3}$	$3.89\cdot10^{-2}$	$1.58\cdot10^{-4}$
14	$1.45\cdot10^{-28}$	$9.40\cdot10^{-2}$	$4.62\cdot10^{-5}$	0.28	0.12	$9.44\cdot10^{-4}$	$3.45\cdot10^{-2}$	$1.33\cdot10^{-4}$
14.5	$1.88\cdot10^{-28}$	$8.98\cdot10^{-2}$	$4.40\cdot10^{-5}$	0.25	0.12	$8.33\cdot10^{-4}$	$2.23\cdot10^{-2}$	$1.60\cdot10^{-4}$
15	$1.70\cdot10^{-28}$	$8.78\cdot10^{-2}$	$4.47\cdot10^{-5}$	0.27	0.11	$7.30\cdot10^{-4}$	$2.18\cdot10^{-2}$	$1.63\cdot10^{-4}$
15.5	$9.94\cdot10^{-23}$	$8.66\cdot10^{-2}$	$4.30\cdot10^{-5}$	0.25	0.11	$6.62\cdot10^{-4}$	$3.67\cdot10^{-3}$	$1.50\cdot10^{-4}$
16	$2.23\cdot10^{-18}$	$7.99\cdot10^{-2}$	$3.78\cdot10^{-5}$	0.20	0.11	$5.83\cdot10^{-4}$	$2.46\cdot10^{-3}$	$1.29\cdot10^{-4}$
16.5	$1.46\cdot10^{-18}$	$7.32\cdot10^{-2}$	$3.52\cdot10^{-5}$	0.21	0.11	$5.01\cdot10^{-4}$	$2.13\cdot10^{-2}$	$1.35\cdot10^{-4}$
17	$1.29\cdot10^{-19}$	$7.51\cdot10^{-2}$	$3.33\cdot10^{-5}$	0.19	0.10	$4.71\cdot10^{-4}$	$1.89\cdot10^{-3}$	$1.60\cdot10^{-4}$
17.5	$1.05\cdot10^{-15}$	$6.98\cdot10^{-2}$	$3.05\cdot10^{-5}$	0.14	0.10	$4.21\cdot10^{-4}$	$5.68\cdot10^{-4}$	$1.25\cdot10^{-4}$
18	$8.45\cdot10^{-16}$	$6.80\cdot10^{-2}$	$2.87\cdot10^{-5}$	0.15	$9.83\cdot10^{-2}$	$3.79\cdot10^{-4}$	$1.28\cdot10^{-3}$	$1.44\cdot10^{-4}$
18.5	$8.66\cdot10^{-16}$	$6.44\cdot10^{-2}$	$2.72\cdot10^{-5}$	0.11	$9.75\cdot10^{-2}$	$3.42\cdot10^{-4}$	$1.19\cdot10^{-3}$	$1.47\cdot10^{-4}$
19	$1.84\cdot10^{-14}$	$6.13\cdot10^{-2}$	$2.63\cdot10^{-5}$	$5.98\cdot10^{-2}$	$9.59\cdot10^{-2}$	$3.08\cdot10^{-4}$	$8.34\cdot10^{-4}$	$1.10\cdot10^{-4}$
19.5	$3.47\cdot10^{-9}$	$5.70\cdot10^{-2}$	$1.45\cdot10^{-5}$	0.18	$9.65\cdot10^{-2}$	$2.68\cdot10^{-4}$	$2.47\cdot10^{-4}$	$1.26\cdot10^{-4}$
20	$2.92\cdot10^{-4}$	$7.96\cdot10^{-2}$	$5.50\cdot10^{-5}$	$8.67\cdot10^{-2}$	$6.97\cdot10^{-2}$	$2.90\cdot10^{-4}$	$1.04\cdot10^{-4}$	$1.59\cdot10^{-4}$

Table S3. Best fit parameters to the generalized Debye model for **2a** in a 1500 Oe dc field.

T / K	$\Delta\chi_S$ / cm^{3}mol^{-1}	χ_T / cm^{3}mol^{-1}	τ / s	α	Residual
6.5	$1.77\cdot10^{-2}$	0.43	0.10	$7.21\cdot10^{-14}$	$4.51\cdot10^{-4}$
7	$1.68\cdot10^{-2}$	0.41	$6.49\cdot10^{-2}$	$1.11\cdot10^{-13}$	$4.21\cdot10^{-4}$
7.5	$1.60\cdot10^{-2}$	0.39	$4.23\cdot10^{-2}$	$1.30\cdot10^{-13}$	$3.18\cdot10^{-4}$
8	$1.57\cdot10^{-2}$	0.37	$2.86\cdot10^{-2}$	$1.32\cdot10^{-13}$	$2.96\cdot10^{-4}$
8.5	$1.48\cdot10^{-2}$	0.35	$1.99\cdot10^{-2}$	$1.26\cdot10^{-13}$	$8.18\cdot10^{-4}$
9	$1.43\cdot10^{-2}$	0.33	$1.44\cdot10^{-2}$	$1.58\cdot10^{-13}$	$2.17\cdot10^{-4}$
9.5	$1.36\cdot10^{-2}$	0.31	$1.07\cdot10^{-2}$	$1.84\cdot10^{-13}$	$2.06\cdot10^{-4}$
10	$1.31\cdot10^{-2}$	0.30	$8.12\cdot10^{-3}$	$2.49\cdot10^{-13}$	$1.73\cdot10^{-4}$
10.5	$1.27\cdot10^{-2}$	0.28	$6.30\cdot10^{-3}$	$2.95\cdot10^{-13}$	$1.95\cdot10^{-4}$
11	$1.22\cdot10^{-2}$	0.27	$4.97\cdot10^{-3}$	$3.51\cdot10^{-13}$	$1.62\cdot10^{-4}$
11.5	$1.21\cdot10^{-2}$	0.26	$3.99\cdot10^{-3}$	$3.82\cdot10^{-13}$	$1.64\cdot10^{-4}$
12	$1.19\cdot10^{-2}$	0.25	$3.24\cdot10^{-3}$	$4.53\cdot10^{-13}$	$1.60\cdot10^{-4}$
12.5	$1.16\cdot10^{-2}$	0.24	$2.66\cdot10^{-3}$	$5.08\cdot10^{-13}$	$1.52\cdot10^{-4}$
13	$1.14\cdot10^{-2}$	0.23	$2.21\cdot10^{-3}$	$5.28\cdot10^{-13}$	$1.84\cdot10^{-4}$
13.5	$1.09\cdot10^{-2}$	0.22	$1.86\cdot10^{-3}$	$5.64\cdot10^{-13}$	$1.86\cdot10^{-4}$
14	$1.10\cdot10^{-2}$	0.21	$1.59\cdot10^{-3}$	$5.59\cdot10^{-13}$	$1.37\cdot10^{-4}$
14.5	$1.06\cdot10^{-2}$	0.21	$1.36\cdot10^{-3}$	$5.81\cdot10^{-13}$	$1.52\cdot10^{-4}$
15	$1.07\cdot10^{-2}$	0.20	$1.17\cdot10^{-3}$	$5.21\cdot10^{-13}$	$1.36\cdot10^{-4}$
15.5	$9.70\cdot10^{-3}$	0.19	$1.01\cdot10^{-3}$	$4.84\cdot10^{-13}$	$1.21\cdot10^{-4}$
16	$1.05\cdot10^{-2}$	0.19	$8.88\cdot10^{-4}$	$4.13\cdot10^{-13}$	$1.38\cdot10^{-4}$
16.5	$1.03\cdot10^{-2}$	0.18	$7.77\cdot10^{-4}$	$4.15\cdot10^{-13}$	$1.31\cdot10^{-4}$
17	$1.02\cdot10^{-2}$	0.18	$6.85\cdot10^{-4}$	$4.08\cdot10^{-13}$	$1.29\cdot10^{-4}$
17.5	$1.05\cdot10^{-2}$	0.17	$6.06\cdot10^{-4}$	$3.14\cdot10^{-13}$	$1.33\cdot10^{-4}$
18	$1.04\cdot10^{-2}$	0.17	$5.40\cdot10^{-4}$	$2.98\cdot10^{-13}$	$1.27\cdot10^{-4}$
18.5	$1.06\cdot10^{-2}$	0.16	$4.78\cdot10^{-4}$	$2.64\cdot10^{-13}$	$1.45\cdot10^{-4}$
19	$1.02\cdot10^{-2}$	0.16	$4.27\cdot10^{-4}$	$2.15\cdot10^{-13}$	$1.38\cdot10^{-4}$
19.5	$1.02\cdot10^{-2}$	0.15	$3.78\cdot10^{-4}$	$1.86\cdot10^{-13}$	$1.46\cdot10^{-4}$
20	$1.01\cdot10^{-2}$	0.15	$3.35\cdot10^{-4}$	$1.77\cdot10^{-13}$	$1.27\cdot10^{-4}$
20.5	$1.15\cdot10^{-2}$	0.15	$3.00\cdot10^{-4}$	$1.46\cdot10^{-13}$	$1.39\cdot10^{-4}$
21	$1.15\cdot10^{-2}$	0.14	$2.64\cdot10^{-4}$	$1.29\cdot10^{-13}$	$2.70\cdot10^{-4}$
21.5	$1.24\cdot10^{-2}$	0.14	$2.33\cdot10^{-4}$	$1.17\cdot10^{-13}$	$1.88\cdot10^{-4}$
22	$6.40\cdot10^{-4}$	0.14	$1.89\cdot10^{-4}$	$2.86\cdot10^{-2}$	$1.25\cdot10^{-3}$

Table S4. Best fit parameters to the generalized Debye model for two processes for **2b** in zero dc field. Due to the poor colecole fit, the data points at 27 K were masked.

T / K	χ_T/ cm^{3}mol^{-1}	$\Delta\chi_{S,FP}$/ cm^{3}mol^{-1}	τ_{FP} / $\cdot$ 10^{-4} s	α_{FP}	$\Delta\chi_{S,SP}$ / cm^{3}mol^{-1}	τ_{SP} / s	α_{SP}	Residual
2	$2.25\cdot10^{-55}$	0.41	4.57	0.42	1.01	2.72	0.55	$3.35\cdot10^{-3}$
3	$1.51\cdot10^{-49}$	0.25	3.43	0.38	0.82	2.50	0.59	$2.89\cdot10^{-4}$
4	$1.19\cdot10^{-35}$	0.21	3.75	0.41	0.56	1.10	0.46	$2.67\cdot10^{-4}$
5	$1.22\cdot10^{-29}$	0.20	5.05	0.46	0.50	0.54	0.27	$2.64\cdot10^{-4}$
6	$6.42\cdot10^{-22}$	0.15	3.66	0.40	0.45	0.20	0.22	$1.04\cdot10^{-4}$
7	$1.17\cdot10^{-21}$	0.13	3.86	0.41	0.33	$7.07\cdot10^{-2}$	0.12	$1.31\cdot10^{-4}$
8	$4.37\cdot10^{-19}$	0.12	3.80	0.40	0.28	$3.44\cdot10^{-2}$	$6.82\cdot10^{-2}$	$1.01\cdot10^{-4}$
9	$1.71\cdot10^{-12}$	0.11	3.66	0.39	0.24	$1.93\cdot10^{-2}$	$4.55\cdot10^{-2}$	$9.43\cdot10^{-5}$

10	$2.25\cdot10^{-12}$	0.10	3.56	0.38	0.22	$1.19\cdot10^{-2}$	$3.30\cdot10^{-2}$	$7.77\cdot10^{-5}$
11	$6.66\cdot10^{-12}$	0.09	3.27	0.36	0.20	$7.76\cdot10^{-3}$	$2.66\cdot10^{-2}$	$8.96\cdot10^{-5}$
12	$5.03\cdot10^{-12}$	0.08	3.07	0.35	0.18	$5.29\cdot10^{-3}$	$2.27\cdot10^{-2}$	$9.17\cdot10^{-5}$
13	$6.07\cdot10^{-12}$	0.07	2.79	0.34	0.17	$3.77\cdot10^{-3}$	$1.96\cdot10^{-2}$	$9.63\cdot10^{-5}$
14	$5.99\cdot10^{-12}$	0.07	2.56	0.31	0.16	$2.79\cdot10^{-3}$	$1.34\cdot10^{-2}$	$9.06\cdot10^{-5}$
15	$7.72\cdot10^{-39}$				0.14	$2.14\cdot10^{-3}$	$4.62\cdot10^{-25}$	$9.47\cdot10^{-5}$
16	$2.21\cdot10^{-38}$				0.13	$1.65\cdot10^{-3}$	$3.32\cdot10^{-25}$	$1.24\cdot10^{-4}$
17	$2.85\cdot10^{-38}$				0.12	$1.32\cdot10^{-3}$	$3.54\cdot10^{-25}$	$1.27\cdot10^{-4}$
18	$4.09\cdot10^{-38}$				0.12	$1.08\cdot10^{-3}$	$3.71\cdot10^{-25}$	$1.05\cdot10^{-4}$
19	$8.58\cdot10^{-38}$				0.12	$8.87\cdot10^{-4}$	$2.18\cdot10^{-25}$	$1.02\cdot10^{-4}$
20	$4.67\cdot10^{-38}$				0.11	$7.51\cdot10^{-4}$	$3.74\cdot10^{-13}$	$9.47\cdot10^{-5}$
21	$4.75\cdot10^{-38}$				0.11	$6.33\cdot10^{-4}$	$1.97\cdot10^{-13}$	$1.14\cdot10^{-4}$
22	$1.64\cdot10^{-37}$				$9.61\cdot10^{-2}$	$5.49\cdot10^{-4}$	$3.40\cdot10^{-14}$	$7.34\cdot10^{-5}$
23	$3.30\cdot10^{-37}$				$9.36\cdot10^{-2}$	$4.71\cdot10^{-4}$	$5.00\cdot10^{-13}$	$8.38\cdot10^{-5}$
24	$2.40\cdot10^{-24}$				$6.04\cdot10^{-2}$	$4.35\cdot10^{-4}$	$1.84\cdot10^{-13}$	$1.08\cdot10^{-4}$
25	$3.73\cdot10^{-16}$				$4.26\cdot10^{-2}$	$3.92\cdot10^{-4}$	$1.98\cdot10^{-3}$	$1.36\cdot10^{-4}$
26	$4.35\cdot10^{-6}$				$3.08\cdot10^{-2}$	$3.35\cdot10^{-4}$	$5.03\cdot10^{-4}$	$9.62\cdot10^{-5}$
27	$2.37\cdot10^{-3}$				$5.45\cdot10^{-3}$	$3.47\cdot10^{-4}$	$4.52\cdot10^{-3}$	$8.95\cdot10^{-5}$

Table S5. Best fit parameters to the generalized Debye model for **2b** in a 1000 Oe dc field.

T / K	$\Delta\chi_S$ / cm^{3}mol^{-1}	χ_T / cm^{3}mol^{-1}	τ / s	α	Residual
7	$1.78\cdot10^{-2}$	$4.62\cdot10^{-1}$	$9.47\cdot10^{-2}$	$5.62\cdot10^{-2}$	$4.87\cdot10^{-4}$
8	$1.59\cdot10^{-2}$	$3.95\cdot10^{-1}$	$4.40\cdot10^{-2}$	$4.01\cdot10^{-2}$	$3.47\cdot10^{-4}$
9	$1.37\cdot10^{-2}$	$3.48\cdot10^{-1}$	$2.36\cdot10^{-2}$	$3.41\cdot10^{-2}$	$2.56\cdot10^{-4}$
10	$1.25\cdot10^{-2}$	$3.14\cdot10^{-1}$	$1.42\cdot10^{-2}$	$3.16\cdot10^{-2}$	$2.75\cdot10^{-4}$
11	$1.13\cdot10^{-2}$	$2.85\cdot10^{-1}$	$9.01\cdot10^{-3}$	$2.72\cdot10^{-2}$	$2.04\cdot10^{-4}$
12	$1.03\cdot10^{-2}$	$2.62\cdot10^{-1}$	$6.06\cdot10^{-3}$	$2.66\cdot10^{-2}$	$1.62\cdot10^{-4}$
13	$9.56\cdot10^{-3}$	$2.41\cdot10^{-1}$	$4.25\cdot10^{-3}$	$2.45\cdot10^{-2}$	$1.69\cdot10^{-4}$
14	$8.85\cdot10^{-3}$	$2.25\cdot10^{-1}$	$3.13\cdot10^{-3}$	$2.09\cdot10^{-2}$	$1.39\cdot10^{-4}$
15	$8.18\cdot10^{-3}$	$2.10\cdot10^{-1}$	$2.36\cdot10^{-3}$	$2.42\cdot10^{-2}$	$1.60\cdot10^{-4}$
16	$7.49\cdot10^{-3}$	$1.98\cdot10^{-1}$	$1.83\cdot10^{-3}$	$2.58\cdot10^{-2}$	$1.28\cdot10^{-4}$
17	$7.52\cdot10^{-3}$	$1.86\cdot10^{-1}$	$1.43\cdot10^{-3}$	$1.94\cdot10^{-2}$	$1.22\cdot10^{-4}$
18	$6.62\cdot10^{-3}$	$1.76\cdot10^{-1}$	$1.16\cdot10^{-3}$	$2.39\cdot10^{-2}$	$1.16\cdot10^{-4}$
19	$5.66\cdot10^{-3}$	$1.67\cdot10^{-1}$	$9.47\cdot10^{-4}$	$2.58\cdot10^{-2}$	$1.15\cdot10^{-4}$
20	$5.49\cdot10^{-3}$	$1.59\cdot10^{-1}$	$7.78\cdot10^{-4}$	$2.05\cdot10^{-2}$	$1.29\cdot10^{-4}$
21	$5.94\cdot10^{-3}$	$1.51\cdot10^{-1}$	$6.51\cdot10^{-4}$	$1.63\cdot10^{-2}$	$1.31\cdot10^{-4}$
22	$3.56\cdot10^{-3}$	$1.45\cdot10^{-1}$	$5.43\cdot10^{-4}$	$3.05\cdot10^{-2}$	$1.07\cdot10^{-4}$
23	$3.45\cdot10^{-3}$	$1.39\cdot10^{-1}$	$4.57\cdot10^{-4}$	$2.94\cdot10^{-2}$	$1.14\cdot10^{-4}$
24	$2.51\cdot10^{-3}$	$1.34\cdot10^{-1}$	$3.81\cdot10^{-4}$	$3.49\cdot10^{-2}$	$7.62\cdot10^{-5}$
25	$8.85\cdot10^{-4}$	$1.29\cdot10^{-1}$	$3.12\cdot10^{-4}$	$3.66\cdot10^{-2}$	$7.01\cdot10^{-5}$
26	$3.02\cdot10^{-15}$	$1.24\cdot10^{-1}$	$2.55\cdot10^{-4}$	$4.03\cdot10^{-2}$	$1.12\cdot10^{-4}$
27	$6.16\cdot10^{-15}$	$1.20\cdot10^{-1}$	$2.07\cdot10^{-4}$	$3.93\cdot10^{-2}$	$1.15\cdot10^{-4}$
28	$7.10\cdot10^{-15}$	$1.15\cdot10^{-1}$	$1.66\cdot10^{-4}$	$3.42\cdot10^{-2}$	$9.49\cdot10^{-5}$

Crystal data

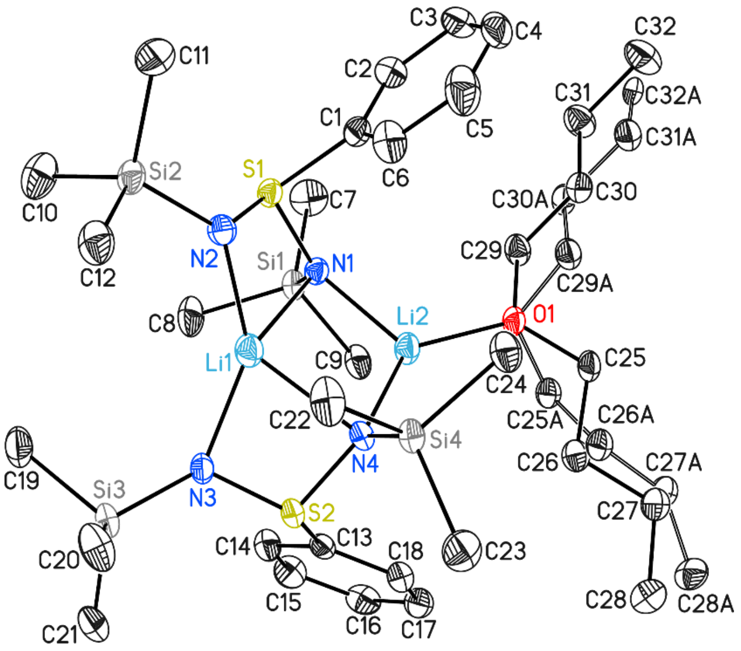

Figure S6. The asymmetric unit of **2_Li**, the anisotropic displacement parameters are depicted at the 50% probability level. Hydrogen atoms are omitted for clarity. The $^{n}Bu_2O$ solvent molecule is disordered over two positions, with an occupancy of 0.9744(19) for the major position. The disorder was refined with distance restraints and restraints for the anisotropic displacement parameters.

Table S6. Crystal data and structure refinement for **2_Li**.

Identification code	2525133	
Empirical formula	$C_{32}H_{64}Li_2N_4OS_2Si_4$	
Formula weight	711.23	
Temperature	100(2) K	
Wavelength	0.71073 Å	
Crystal system	Monoclinic	
Space group	*P*2$_1$/*c*	
Unit cell dimensions	a = 23.620(3) Å	a= 90°.
	b = 10.571(2) Å	b= 112.78(2)°
	c = 18.775(3) Å	g = 90°.
Volume	4322.2(13) Å^3	
Z	4	
Density (calculated)	1.093 Mg/m^3	
Absorption coefficient	0.262 mm^{-1}	

F(000)	1544
Crystal size	0.238 x 0.232 x 0.064 mm^3
Theta range for data collection	0.935 to 26.475°.
Index ranges	-29<=h<=29, -13<=k<=13, -23<=l<=23
Reflections collected	142441
Independent reflections	8875 [R_{int} = 0.0661]
Completeness to theta = 25.242°	100.0 %
Absorption correction	Semi-empirical from equivalents
Max. and min. transmission	0.7454 and 0.6885
Refinement method	Full-matrix least-squares on F^2
Data / restraints / parameters	8875 / 355 / 493
Goodness-of-fit on F^2	1.064
Final R indices [$I > 2\sigma(I)$]	R_1 = 0.0372, wR_2 = 0.0811
R indices (all data)	R_1 = 0.0567, wR_2 = 0.0908
Largest diff. peak and hole	0.288 and -0.373 eÅ^{-3}

Table S7. Bond lengths [Å] and angles [°] for **3**.

S(1)-N(2)	1.5935(16)	N(1)-Si(1)	1.7373(16)
S(1)-N(1)	1.6266(15)	N(1)-Li(1)	2.259(4)
S(1)-C(1)	1.810(2)	C(1)-C(6)	1.375(3)
S(1)-Li(1)	2.658(3)	Li(1)-N(3)	1.991(4)
Li(2)-O(1)	1.948(3)	Li(1)-N(4)	2.245(4)
Li(2)-N(1)	2.034(3)	Li(1)-S(2)	2.636(3)
Li(2)-N(4)	2.038(3)	Si(1)-C(9)	1.865(2)
Li(2)-Li(1)	2.670(4)	Si(1)-C(8)	1.868(2)
Li(2)-C(25A)	2.76(5)	Si(1)-C(7)	1.868(2)
Li(2)-Si(1)	3.159(3)	S(2)-N(3)	1.6005(15)
Li(2)-Si(4)	3.186(3)	S(2)-N(4)	1.6329(15)
C(2)-C(1)	1.388(3)	S(2)-C(13)	1.8034(19)
C(2)-C(3)	1.388(3)	C(3)-C(4)	1.374(3)
Si(2)-N(2)	1.7189(16)	Si(3)-N(3)	1.7238(16)
Si(2)-C(12)	1.856(2)	Si(3)-C(19)	1.861(2)
Si(2)-C(10)	1.865(2)	Si(3)-C(20)	1.869(2)
Si(2)-C(11)	1.869(2)	Si(3)-C(21)	1.875(2)
N(2)-Li(1)	1.970(4)	C(4)-C(5)	1.382(3)

Si(4)-N(4)	1.7378(16)
Si(4)-C(23)	1.866(2)
Si(4)-C(24)	1.867(2)
Si(4)-C(22)	1.868(2)
C(5)-C(6)	1.388(3)
C(13)-C(14)	1.385(3)
C(13)-C(18)	1.391(2)
C(14)-C(15)	1.388(3)
C(15)-C(16)	1.381(3)
C(16)-C(17)	1.384(3)
C(17)-C(18)	1.389(3)
O(1)-C(29A)	1.424(16)
O(1)-C(25A)	1.434(16)
O(1)-C(25)	1.437(2)
O(1)-C(29)	1.451(2)
C(25)-C(26)	1.501(3)
C(26)-C(27)	1.523(3)
C(27)-C(28)	1.514(3)
C(29)-C(30)	1.508(3)
C(30)-C(31)	1.525(3)
C(31)-C(32)	1.516(3)
C(25A)-C(26A)	1.502(18)
C(26A)-C(27A)	1.521(18)
C(27A)-C(28A)	1.515(18)
C(29A)-C(30A)	1.509(17)
C(30A)-C(31A)	1.517(17)
C(31A)-C(32A)	1.515(17)

N(2)-S(1)-N(1)	104.90(8)
N(2)-S(1)-C(1)	104.03(9)
N(1)-S(1)-C(1)	102.40(8)
N(2)-S(1)-Li(1)	47.47(9)
N(1)-S(1)-Li(1)	57.79(9)
C(1)-S(1)-Li(1)	117.18(10)
O(1)-Li(2)-N(1)	114.41(16)
O(1)-Li(2)-N(4)	134.93(17)
N(1)-Li(2)-N(4)	110.32(15)
O(1)-Li(2)-Li(1)	167.39(18)
N(1)-Li(2)-Li(1)	55.42(11)
N(4)-Li(2)-Li(1)	54.98(11)
O(1)-Li(2)-C(25A)	29.5(10)
N(1)-Li(2)-C(25A)	131.7(10)
N(4)-Li(2)-C(25A)	114.4(10)
Li(1)-Li(2)-C(25A)	162.6(10)
O(1)-Li(2)-Si(1)	102.60(12)
N(1)-Li(2)-Si(1)	30.29(7)
N(4)-Li(2)-Si(1)	119.08(13)
Li(1)-Li(2)-Si(1)	72.13(10)
C(25A)-Li(2)-Si(1)	107.5(10)
O(1)-Li(2)-Si(4)	114.19(13)
N(1)-Li(2)-Si(4)	121.51(13)
N(4)-Li(2)-Si(4)	29.66(6)
Li(1)-Li(2)-Si(4)	71.04(10)
C(25A)-Li(2)-Si(4)	106.5(10)
Si(1)-Li(2)-Si(4)	142.99(10)
C(1)-C(2)-C(3)	119.5(2)
N(2)-Si(2)-C(12)	105.49(9)
N(2)-Si(2)-C(10)	113.13(10)
C(12)-Si(2)-C(10)	108.81(11)
N(2)-Si(2)-C(11)	112.35(9)
C(12)-Si(2)-C(11)	109.88(11)
C(10)-Si(2)-C(11)	107.14(11)
S(1)-N(2)-Si(2)	121.58(10)
S(1)-N(2)-Li(1)	95.93(12)
Si(2)-N(2)-Li(1)	133.60(13)
S(1)-N(1)-Si(1)	115.10(9)
S(1)-N(1)-Li(2)	131.07(13)
Si(1)-N(1)-Li(2)	113.52(12)
S(1)-N(1)-Li(1)	84.68(10)
Si(1)-N(1)-Li(1)	119.04(11)
Li(2)-N(1)-Li(1)	76.72(13)
C(6)-C(1)-C(2)	120.41(19)
C(6)-C(1)-S(1)	121.48(16)
C(2)-C(1)-S(1)	118.09(16)
N(2)-Li(1)-N(3)	143.61(18)
N(2)-Li(1)-N(4)	131.94(17)
N(3)-Li(1)-N(4)	75.30(12)
N(2)-Li(1)-N(1)	73.92(12)

N(3)-Li(1)-N(1)	134.34(18)	C(20)-Si(3)-C(21)	106.76(10)
N(4)-Li(1)-N(1)	95.83(13)	C(3)-C(4)-C(5)	120.0(2)
N(2)-Li(1)-S(2)	159.92(17)	N(4)-Si(4)-C(23)	112.29(9)
N(3)-Li(1)-S(2)	37.30(7)	N(4)-Si(4)-C(24)	107.70(8)
N(4)-Li(1)-S(2)	38.04(6)	C(23)-Si(4)-C(24)	107.91(10)
N(1)-Li(1)-S(2)	119.44(13)	N(4)-Si(4)-C(22)	111.67(9)
N(2)-Li(1)-S(1)	36.60(7)	C(23)-Si(4)-C(22)	108.27(11)
N(3)-Li(1)-S(1)	157.41(17)	C(24)-Si(4)-C(22)	108.89(10)
N(4)-Li(1)-S(1)	121.59(13)	N(4)-Si(4)-Li(2)	35.46(8)
N(1)-Li(1)-S(1)	37.53(6)	C(23)-Si(4)-Li(2)	127.50(9)
S(2)-Li(1)-S(1)	155.49(13)	C(24)-Si(4)-Li(2)	72.37(9)
N(2)-Li(1)-Li(2)	105.49(15)	C(22)-Si(4)-Li(2)	121.46(9)
N(3)-Li(1)-Li(2)	110.85(16)	C(4)-C(5)-C(6)	120.2(2)
N(4)-Li(1)-Li(2)	48.03(10)	C(1)-C(6)-C(5)	119.6(2)
N(1)-Li(1)-Li(2)	47.86(10)	S(2)-N(3)-Si(3)	119.27(9)
S(2)-Li(1)-Li(2)	78.22(11)	S(2)-N(3)-Li(1)	93.77(11)
S(1)-Li(1)-Li(2)	77.53(11)	Si(3)-N(3)-Li(1)	135.38(12)
N(1)-Si(1)-C(9)	106.97(8)	S(2)-N(4)-Si(4)	113.73(9)
N(1)-Si(1)-C(8)	110.80(9)	S(2)-N(4)-Li(2)	131.21(13)
C(9)-Si(1)-C(8)	108.95(10)	Si(4)-N(4)-Li(2)	114.88(12)
N(1)-Si(1)-C(7)	112.93(9)	S(2)-N(4)-Li(1)	84.06(10)
C(9)-Si(1)-C(7)	108.80(10)	Si(4)-N(4)-Li(1)	118.27(11)
C(8)-Si(1)-C(7)	108.31(11)	Li(2)-N(4)-Li(1)	76.99(13)
N(1)-Si(1)-Li(2)	36.20(8)	C(14)-C(13)-C(18)	120.08(17)
C(9)-Si(1)-Li(2)	70.78(9)	C(14)-C(13)-S(2)	120.93(14)
C(8)-Si(1)-Li(2)	122.84(10)	C(18)-C(13)-S(2)	118.85(14)
C(7)-Si(1)-Li(2)	126.30(10)	C(13)-C(14)-C(15)	119.93(18)
N(3)-S(2)-N(4)	106.76(8)	C(16)-C(15)-C(14)	120.03(19)
N(3)-S(2)-C(13)	103.96(8)	C(15)-C(16)-C(17)	120.23(18)
N(4)-S(2)-C(13)	102.17(8)	C(16)-C(17)-C(18)	120.05(18)
N(3)-S(2)-Li(1)	48.93(9)	C(17)-C(18)-C(13)	119.66(18)
N(4)-S(2)-Li(1)	57.90(9)	C(29A)-O(1)-C(25A)	117(3)
C(13)-S(2)-Li(1)	109.91(9)	C(25)-O(1)-C(29)	111.38(13)
C(4)-C(3)-C(2)	120.3(2)	C(29A)-O(1)-Li(2)	131(2)
N(3)-Si(3)-C(19)	106.38(9)	C(25A)-O(1)-Li(2)	109(3)
N(3)-Si(3)-C(20)	113.06(9)	C(25)-O(1)-Li(2)	131.06(14)
C(19)-Si(3)-C(20)	109.49(11)	C(29)-O(1)-Li(2)	117.18(14)
N(3)-Si(3)-C(21)	112.58(9)	O(1)-C(25)-C(26)	111.18(15)
C(19)-Si(3)-C(21)	108.49(10)	C(25)-C(26)-C(27)	111.35(17)

C(28)-C(27)-C(26)	113.71(19)	C(26A)-C(25A)-Li(2)	106(3)
O(1)-C(29)-C(30)	114.76(16)	C(25A)-C(26A)-C(27A)	112(3)
C(29)-C(30)-C(31)	110.77(17)	C(28A)-C(27A)-C(26A)	114(3)
C(32)-C(31)-C(30)	113.39(18)	O(1)-C(29A)-C(30A)	110(2)
O(1)-C(25A)-C(26A)	114(2)	C(29A)-C(30A)-C(31A)	111(2)
O(1)-C(25A)-Li(2)	41.9(16)	C(32A)-C(31A)-C(30A)	113(2)

High resolution XRD and multipole modelling

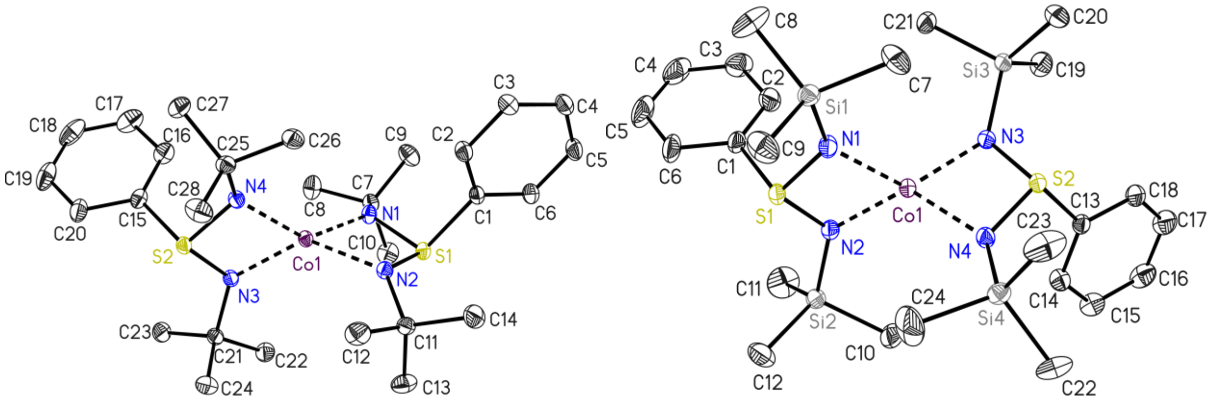

Figure S7. Complex **1_Co** and **2_Co**. Displacement parameters are depicted at a 50% probability level. Hydrogen atoms are omitted for clarity.

Table S8. Constraints in the multipole refinement.

**1_Co**		**2_Co**		
Atom	Chemical constraints	Atom	Chemical constraints	Local symmetry
Co(1)		Co(1)		No
S(1)	S(2)	S(1)	S(2)	C_s
N(1)	N(2), N(3), N(4)	N(1)	N(2), N(3), N(4)	No
C(1)	C(15)	C(1)	C(13)	C_{2v}
C(2)	C(6), C(16), C(20)	C(2)	C(6), C(14), C(18)	C_{2v}
C(3)	C(5), C(17), C(19)	C(3)	C(5), C(15), C(17)	C_{2v}
C(4)	C(18)	C(4)	C(16)	C_{2v}
C(7)	C(11), C(21), C(25)	Si(1)	Si(2), Si(3), Si(4)	C_{3v}
C(8)	C(9), C(10), C(12), C(13), C(14), C(22), C(23), C(24), C(26), C(27), C(28)	C(7), C(8) C(9), C(10), C(11), C(12), C(19), C(20), C(21), C(22), C(23), C(24)		C_{3v}
H(2)	H(6), H(16), H(20)	H(2)	H(6), H(14), H(18)	C_∞
H(3)	H(5), H(17), H(19)	H(3)	H(5), H(15), H(17)	C_∞
H(4)	H(18)	H(4)	H(16)	C_∞
H(8A)	H(8B), H(8C), H(9A), H(9B), H(9C), H(10A), H(10B), H(10C), H(12A), H(12B), H(12C), H(13A), H(13B), H(13C), H(14A), H(14B), H(14C), H(22A), H(22B), H(22C), H(23A), H(23B), H(23C), H(24A),	H(7A)	H(7B), H(7C), H(8A), H(8B), H(8C), H(9A), H(9B), H(9C), H(10A), H(10B), H(10C), H(11A), H(11B), H(11C), H(12A), H(12B), H(12C), H(19A), H(19B), H(19C), H(20A), H(20B), H(20C), H(21A),	C_∞

	H(24B), H(24C), H(26A), H(26B), H(26C), H(27A), H(27B), H(27C), H(28A), H(28B), H(28C)		H(21B), H(21C), H(22A), H(22B), H(22C), H(23A), H(23B), H(23C), H(24A), H(24B), H(24C)	

Table S9. Refinement steps of the multipole refinement.

Step	Refined parameters
1	Scaling
2	+ Monopoles
3	+ Multipoles up to hexadecapoles (H: quadrupoles)
4	+ anisotropic displacement
5	+ positions of non-hydrogen atoms
6	+ κ of non-hydrogen atoms
7	Hydrogen atom positions
8	as step 6
9	κ' of non-hydrogen atoms
10	as step 6
11	+ including weak reflections
12	reducing the symmetry of nitrogen atoms
13	+ Gram Charlier parameters of 3rd order
14	+ quadrupoles at hydrogen atoms
15	κ' of non-hydrogen atoms
16	as step 14

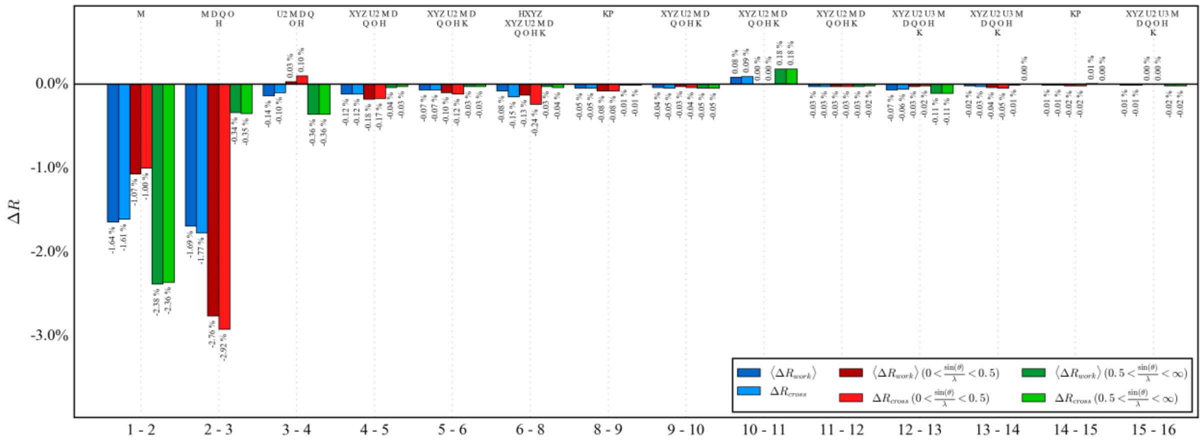

Figure S8. Cross validation[62] of **1_Co**.

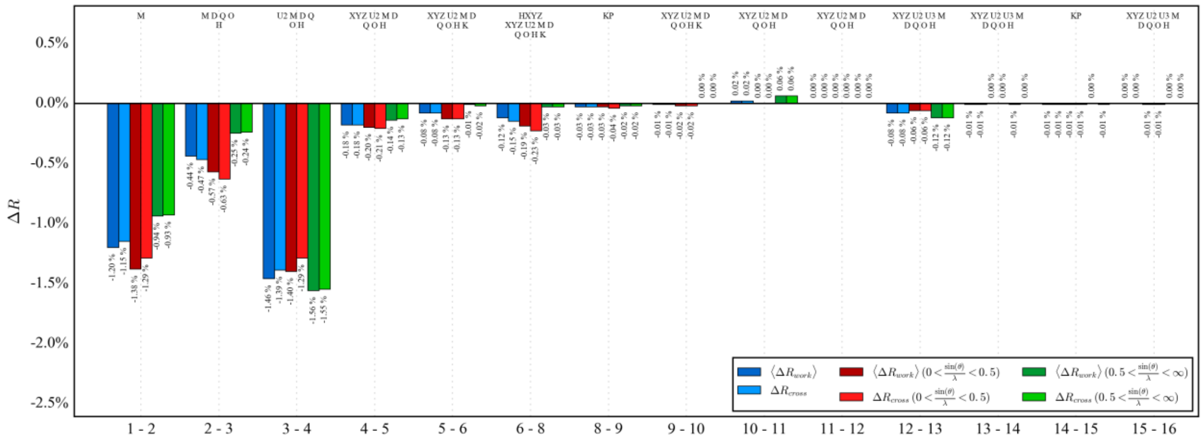

Figure S9. Cross validation[62] of **2_Co**.

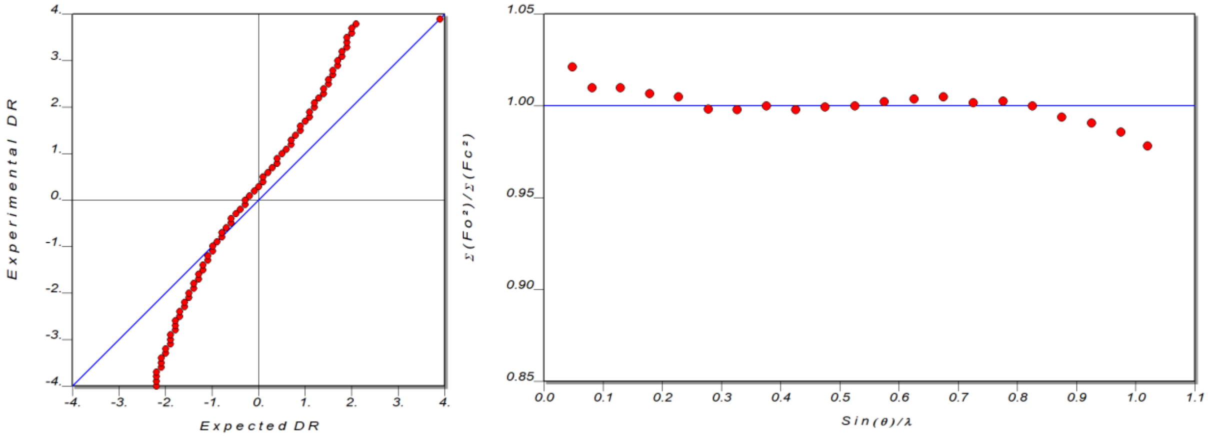

Figure S10. Normal probability plot[63] and DRK plot[11] **of 1_Co**.

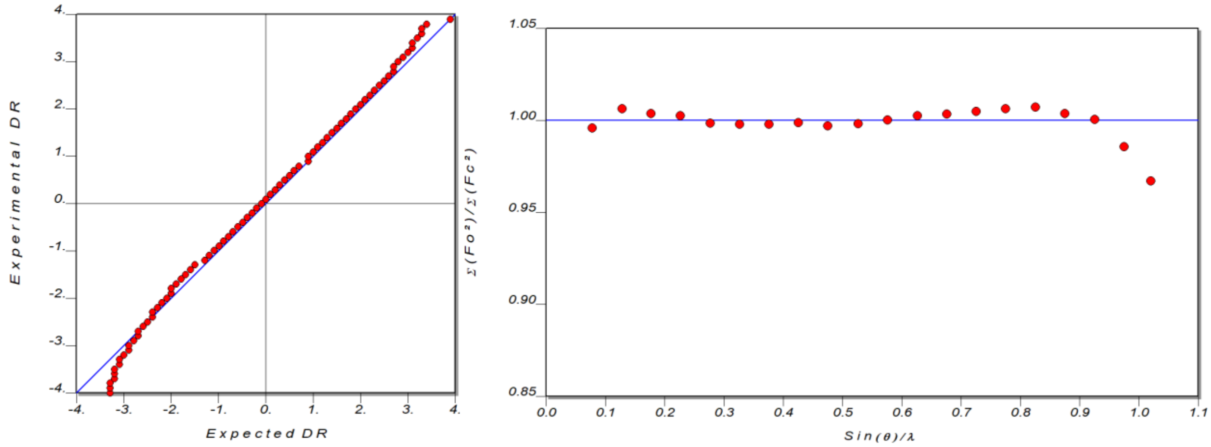

Figure S11. Normal probability plot[63] and DRK plot[64] of **2_Co**.

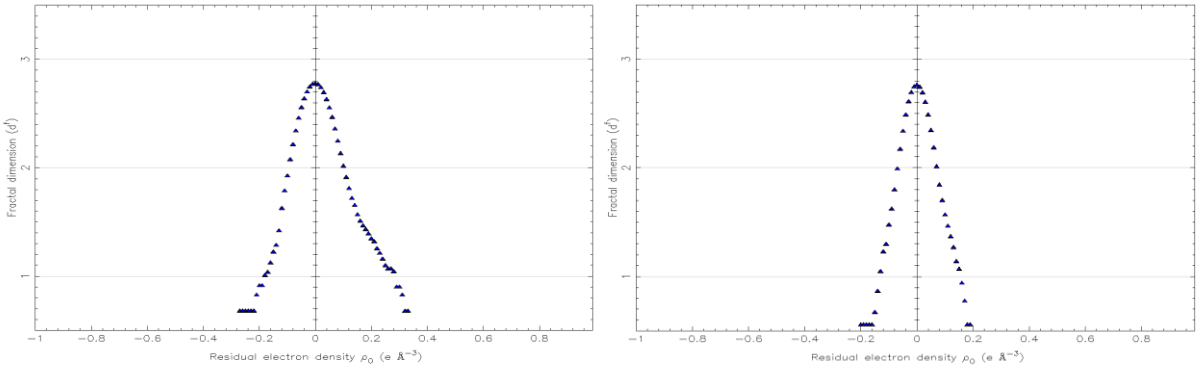

Figure S12. Henn-Meindl Plots[65] of **1_Co** (left) and **2_Co** (right).

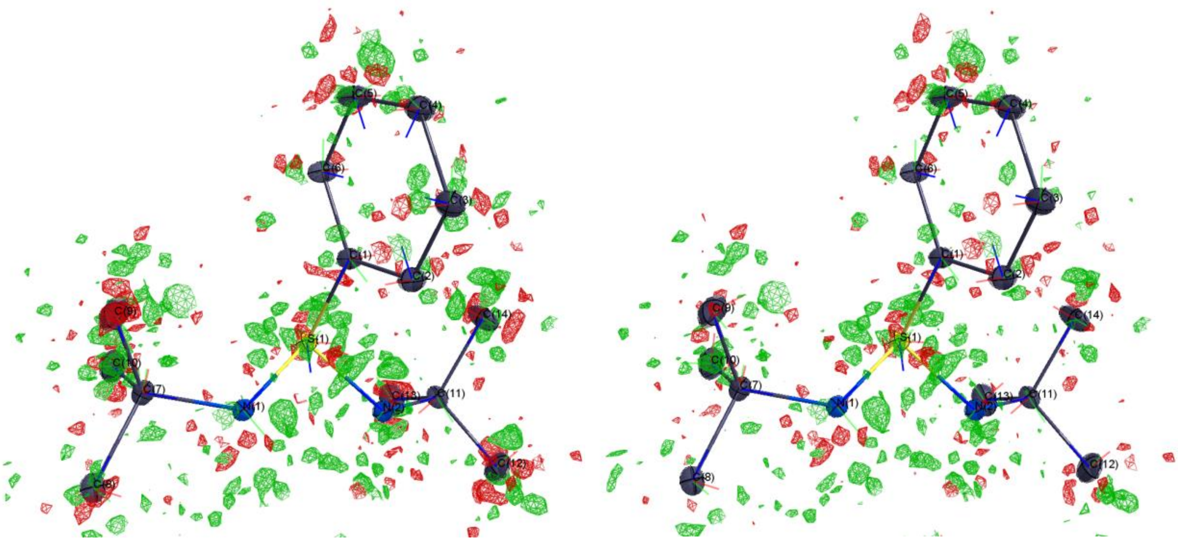

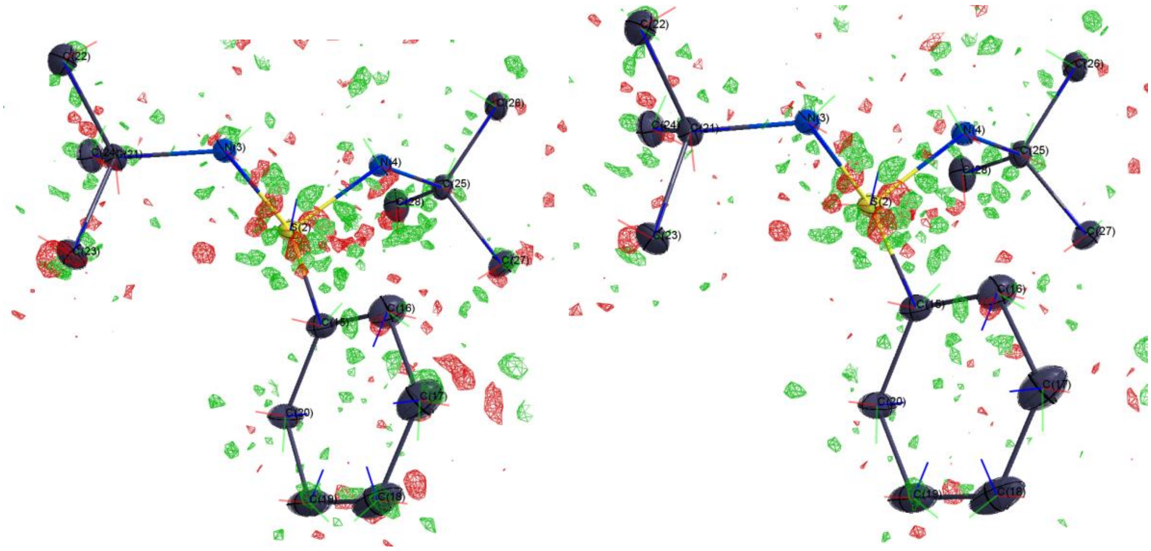

Figure S13. 1_Co before (left) and after (right) refinement of third order Gram-Charlier parameters. Isolevel: 0.1061e/A^3

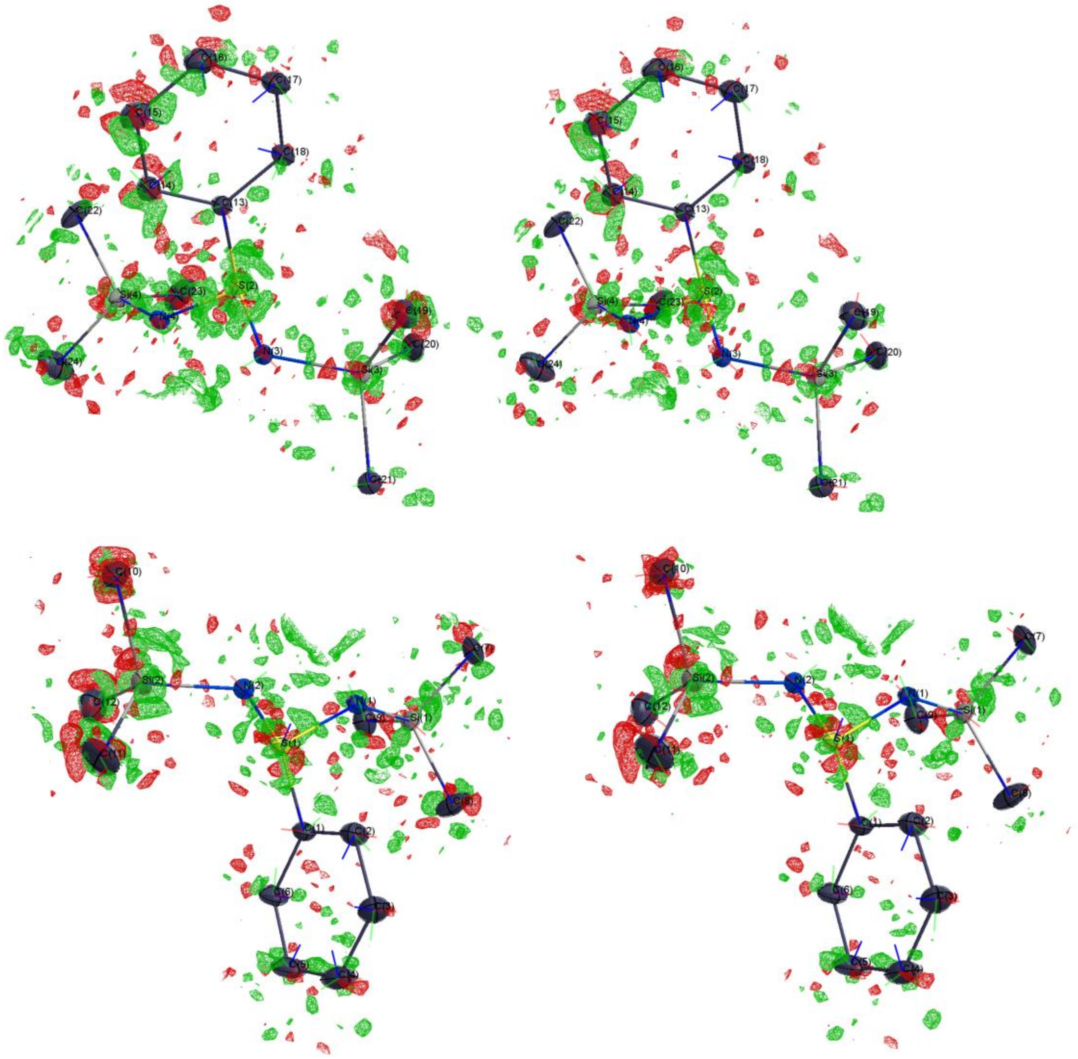

Figure S14. 2_Co before (left) and after (right) refinement of third order Gram-Charlier parameters. Isolevel: 0.0624e/A^3

Table S10. Crystal data and structure refinement for **1_Co** and **2_Co**.

	**1_Co**	**2_Co**
CCDC number	2525131	2525132
Formula	$C_{28}H_{46}CoN_4S_2$	$C_{24}H_{46}CoN_4S_2Si_4$
Formula weight	561.74	626.06
T [K]	100(2)	100(2)
λ (Å)	0.71073	0.71073
Crystal system	Monoclinic	Monoclinic
Space group	$P2_1/c$	$P2_1$
a (Å)	9.054(2)	9.659(2)
b (Å)	10.987(2)	11.378(2)
c (Å)	30.806(3)	15.600(3)
β (°)	91.83(2)	93.50(2)
Volume (Å^3)	3062.9(9)	1711.2(6)
Z	4	2
μ (mm^{-1})	0.719	0.783
F(000)	1204	666
Crystal size [mm]	0.308 x 0.205 x 0.178	0.234 x 0.345 x 0.358
Θ range [°]	1.325 to 47.66	1.308 to 46.66
Reflections collected	544098	457296
Independent reflections	29002	32374
R_{int}	0.0349	0.0438
**IAM Refinement**		
Data / restraints / parameters	29002 / 0 / 328	32374 / 1 / 328
$R1$ [$I > 2\sigma(I)$]	0.0236	0.0170
$wR2$ [all data]	0.0719	0.0445
$\Delta\rho_{max}$, $\Delta\rho_{min}$ (e Å^{-3})	0.570, -0.353	0.425, -0.239
**Multipole Refinement**		
Data / parameters	49.5241	61.2950
R_1(F^2)	0.0162	0.0137
GOF	1.777	0.9974
$\Delta\rho_{max}$, $\Delta\rho_{min}$ (e Å^{-3})	0.334, -0.276	0.202, -0.186

Table S11. Properties of **1_Co** and the second dataset of **1_Co**.

	1_Co paper		1_Co second measurement	
Geometry				
Co - N	2.0112(2)		2.0110(2)	
S - N<br>C - S<br>C/Si - N<br>N - Co - N<br>N - S - N<br>S - N - C/S	1.6344(2)<br>1.8040(3)<br>1.4774(3)<br>72.755(9)<br>93.74(2)<br>119.03(2)		1.6336(2)<br>1.8035(2)<br>1.4776(2)<br>72.720(7)<br>93.743(8)<br>119.04(2)	
Bond Critical Points (mean values)				
	ρ	ε	ρ	ε
Co - N<br>S - N<br>C/Si - N<br>C - S	0.61<br>1.69<br>1.76<br>1.32	0.14<br>0.15<br>0.04<br>0.04	0.57<br>1.60<br>1.73<br>1.28	0.17<br>0.23<br>0.04<br>0.10
	$\nabla^2\rho$	|V|/G	$\nabla^2\rho$	|V|/G
Co - N<br>S - N<br>C/Si - N<br>C - S	9.17<br>-9.17<br>-13.64<br>-5.91	1.18<br>2.43<br>2.67<br>2.43	9.63<br>-5.41<br>-12.10<br>-4.19	1.11<br>2.25<br>2.59<br>2.29
Bader Charges (mean values)				
Co<br>N<br>S<br>C/Si<br>C (tBu/tms)<br>C1 (Ph)<br>C2 (Ph)<br>C3 (Ph)<br>C4 (Ph)	0.92<br>-1.14<br>0.97<br>0.49<br>0.08<br>-0.16<br>-0.17<br>-0.13<br>-0.16		0.97<br>-1.13<br>0.99<br>0.41<br>0.04<br>-0.12<br>-0.16<br>-0.13<br>-0.15	
d-orbital populations				
z^2<br>xz<br>yz<br>$x^2{-}y^2$<br>xy<br>Total 3d<br>4s	1.82(2) (25.5 %)<br>1.12(2) (15.7 %)<br>1.15(2) (16.0 %)<br>1.37(2) (19.1 %)<br>1.69(2) (23.7 %)<br>7.15(2)<br>1.33(3)		1.71(1) (23.5 %)<br>1.18(1) (16.2 %)<br>1.08(1) (14.8 %)<br>1.55(1) (21.2 %)<br>1.78(1) (24.4 %)<br>7.30(2)<br>1.24(4)	

Table S12. BCP properties of **1_Co**

A - B	d1	d2	f	del2f	ell	Gb	Gb/f	Vb	Eb
CO(1) -N(1)	0.9932	1.0152	0.62	9.22	0.17	0.79	1.28	-0.94	-0.15
CO(1) -N(2)	0.9903	1.0233	0.6	9.13	0.12	0.77	1.28	-0.9	-0.13
CO(1) -N(3)	0.9974	1.0167	0.61	9.02	0.11	0.78	1.27	-0.93	-0.15
CO(1) -N(4)	0.9852	1.0239	0.6	9.32	0.15	0.78	1.3	-0.9	-0.12
S(1) -N(1)	0.7862	0.8489	1.69	-9.18	0.15	1.49	0.89	-3.63	-2.14
S(1) -N(2)	0.7857	0.849	1.69	-9.21	0.15	1.49	0.88	-3.63	-2.14

C(1) -S(1)	0.8898	0.9133	1.32	-5.93	0.04	1	0.76	-2.43	-1.42
C(7) -N(1)	0.6319	0.8469	1.75	-13.53	0.03	1.42	0.81	-3.78	-2.36
C(11) -N(2)	0.6306	0.8466	1.76	-13.64	0.04	1.42	0.81	-3.8	-2.38
S(2) -N(3)	0.7883	0.8492	1.68	-9.04	0.14	1.49	0.89	-3.61	-2.12
S(2) -N(4)	0.7851	0.8489	1.69	-9.25	0.15	1.49	0.88	-3.63	-2.14
C(15) -S(2)	0.8905	0.9143	1.32	-5.88	0.04	1	0.76	-2.42	-1.41
C(21) -N(3)	0.6301	0.8471	1.76	-13.69	0.04	1.42	0.81	-3.8	-2.38
C(25) -N(4)	0.6299	0.8471	1.76	-13.69	0.03	1.42	0.81	-3.8	-2.38
C(1) -C(2)	0.6933	0.7	2.18	-20.09	0.17	2	0.92	-5.41	-3.41
C(1) -C(6)	0.6928	0.6994	2.18	-20.22	0.18	2	0.92	-5.43	-3.42
H(2) -C(2)	0.3504	0.7326	1.78	-18.91	0.1	1.23	0.69	-3.78	-2.55
H(4) -C(4)	0.3566	0.7263	1.79	-18.92	0.03	1.24	0.69	-3.81	-2.57
H(6) -C(6)	0.3505	0.7326	1.78	-18.91	0.1	1.23	0.69	-3.78	-2.55
C(3) -C(2)	0.6905	0.7015	2.17	-20.04	0.14	1.99	0.92	-5.38	-3.39
H(3) -C(3)	0.3495	0.7335	1.73	-16.66	0.05	1.23	0.71	-3.63	-2.4
H(5) -C(5)	0.3496	0.7334	1.73	-16.65	0.05	1.23	0.71	-3.62	-2.39
C(3) -C(4)	0.6916	0.7013	2.16	-19.8	0.17	1.97	0.91	-5.32	-3.36
C(5) -C(4)	0.6903	0.7001	2.16	-19.95	0.17	1.98	0.91	-5.35	-3.37
C(5) -C(6)	0.6936	0.7047	2.15	-19.55	0.14	1.97	0.92	-5.31	-3.34
C(7) -C(8)	0.7407	0.79	1.69	-15.17	0.03	1.22	0.72	-3.5	-2.28
C(7) -C(9)	0.7429	0.7914	1.68	-15.06	0.04	1.21	0.72	-3.48	-2.27
C(7) -C(10)	0.7421	0.7914	1.68	-15.02	0.04	1.22	0.72	-3.48	-2.27
H(8A) -C(8)	0.4055	0.6716	1.75	-18.09	0.06	1.2	0.69	-3.67	-2.47
H(8B) -C(8)	0.4051	0.6719	1.75	-18.06	0.06	1.2	0.69	-3.67	-2.47
H(8C) -C(8)	0.4051	0.672	1.75	-18.08	0.06	1.2	0.69	-3.67	-2.47
H(9A) -C(9)	0.4055	0.6716	1.75	-18.08	0.06	1.2	0.69	-3.66	-2.46
H(9B) -C(9)	0.4049	0.6722	1.75	-18.05	0.06	1.21	0.69	-3.68	-2.47
H(9C) -C(9)	0.405	0.6719	1.75	-18.08	0.06	1.2	0.69	-3.67	-2.47
H(10A) -C(10)	0.4057	0.6714	1.75	-18.07	0.06	1.2	0.68	-3.66	-2.46
H(10B) -C(10)	0.405	0.6721	1.75	-18.03	0.06	1.21	0.69	-3.68	-2.47
H(10C) -C(10)	0.4054	0.6716	1.75	-18.07	0.06	1.2	0.69	-3.66	-2.46
C(11) -C(12)	0.7386	0.7872	1.7	-15.4	0.03	1.23	0.72	-3.54	-2.31
C(11) -C(13)	0.7399	0.789	1.69	-15.26	0.04	1.22	0.72	-3.52	-2.29
C(11) -C(14)	0.7418	0.7904	1.69	-15.12	0.04	1.22	0.72	-3.5	-2.28
H(12A) -C(12)	0.4048	0.6722	1.75	-18.07	0.06	1.21	0.69	-3.68	-2.47
H(12B) -C(12)	0.4051	0.6719	1.75	-18.07	0.06	1.2	0.69	-3.67	-2.47
H(12C) -C(12)	0.4051	0.6718	1.75	-18.08	0.06	1.2	0.69	-3.67	-2.47
H(13A) -C(13)	0.405	0.672	1.75	-18.08	0.06	1.21	0.69	-3.68	-2.47
H(13B) -C(13)	0.4054	0.6716	1.75	-18.07	0.06	1.2	0.69	-3.66	-2.46
H(13C) -C(13)	0.4052	0.6718	1.75	-18.08	0.06	1.2	0.69	-3.67	-2.47
H(14A) -C(14)	0.4054	0.6716	1.75	-18.08	0.06	1.2	0.69	-3.66	-2.47
H(14B) -C(14)	0.405	0.672	1.75	-18.07	0.06	1.21	0.69	-3.68	-2.47
H(14C) -C(14)	0.4053	0.6717	1.75	-18.08	0.06	1.2	0.69	-3.67	-2.47
C(15) -C(16)	0.6942	0.701	2.17	-19.94	0.17	1.99	0.92	-5.38	-3.39
C(15) -C(20)	0.6929	0.6993	2.18	-20.22	0.18	2	0.92	-5.42	-3.42
H(16) -C(16)	0.3505	0.7326	1.78	-18.9	0.1	1.23	0.69	-3.78	-2.55
H(18) -C(18)	0.3591	0.7239	1.74	-15.99	0.03	1.27	0.73	-3.66	-2.39

H(20)	-C(20)	0.3505	0.7325	1.78	-18.93	0.1	1.23	0.69	-3.78	-2.55
C(17)	-C(16)	0.6904	0.7016	2.17	-20.05	0.14	1.99	0.92	-5.38	-3.39
H(17)	-C(17)	0.3496	0.7335	1.73	-16.63	0.05	1.23	0.71	-3.62	-2.39
H(19)	-C(19)	0.3496	0.7334	1.73	-16.66	0.05	1.23	0.71	-3.63	-2.4
C(17)	-C(18)	0.6938	0.7017	2.15	-19.62	0.18	1.95	0.91	-5.28	-3.33
C(19)	-C(18)	0.6894	0.6974	2.17	-20.26	0.17	1.98	0.91	-5.38	-3.4
C(19)	-C(20)	0.6941	0.7052	2.15	-19.47	0.14	1.97	0.92	-5.3	-3.33
C(21)	-C(22)	0.7408	0.79	1.69	-15.19	0.03	1.22	0.72	-3.5	-2.28
C(21)	-C(23)	0.743	0.792	1.68	-14.99	0.04	1.21	0.72	-3.47	-2.26
C(21)	-C(24)	0.7439	0.7931	1.68	-14.93	0.04	1.21	0.72	-3.46	-2.25
H(22A)	-C(22)	0.4053	0.6717	1.75	-18.08	0.06	1.2	0.69	-3.67	-2.47
H(22B)	-C(22)	0.4051	0.6719	1.75	-18.08	0.06	1.2	0.69	-3.67	-2.47
H(22C)	-C(22)	0.4051	0.672	1.75	-18.08	0.06	1.2	0.69	-3.67	-2.47
H(23A)	-C(23)	0.405	0.672	1.75	-18.08	0.06	1.21	0.69	-3.68	-2.47
H(23B)	-C(23)	0.4054	0.6716	1.75	-18.09	0.06	1.2	0.69	-3.67	-2.47
H(23C)	-C(23)	0.4056	0.6714	1.75	-18.07	0.06	1.2	0.68	-3.66	-2.46
H(24A)	-C(24)	0.4052	0.6718	1.75	-18.08	0.06	1.2	0.69	-3.67	-2.47
H(24B)	-C(24)	0.4053	0.6717	1.75	-18.08	0.06	1.2	0.69	-3.67	-2.47
H(24C)	-C(24)	0.4056	0.6714	1.75	-18.08	0.06	1.2	0.68	-3.66	-2.46
C(25)	-C(26)	0.7411	0.7902	1.69	-15.18	0.03	1.22	0.72	-3.5	-2.28
C(25)	-C(27)	0.7431	0.7918	1.68	-15.03	0.04	1.21	0.72	-3.47	-2.26
C(25)	-C(28)	0.7422	0.7912	1.69	-15.05	0.04	1.22	0.72	-3.49	-2.27
H(26A)	-C(26)	0.405	0.672	1.75	-18.08	0.06	1.2	0.69	-3.67	-2.47
H(26B)	-C(26)	0.4053	0.6717	1.75	-18.09	0.06	1.2	0.69	-3.67	-2.47
H(26C)	-C(26)	0.4053	0.6717	1.75	-18.08	0.06	1.2	0.69	-3.67	-2.47
H(27A)	-C(27)	0.4049	0.6721	1.75	-18.06	0.06	1.2	0.69	-3.67	-2.47
H(27B)	-C(27)	0.4055	0.6715	1.75	-18.08	0.06	1.2	0.69	-3.66	-2.46
H(27C)	-C(27)	0.405	0.672	1.75	-18.07	0.06	1.21	0.69	-3.68	-2.47
H(28A)	-C(28)	0.4057	0.6713	1.75	-18.09	0.06	1.2	0.68	-3.66	-2.46
H(28B)	-C(28)	0.405	0.672	1.75	-18.06	0.06	1.21	0.69	-3.68	-2.47
H(28C)	-C(28)	0.4053	0.6718	1.75	-18.09	0.06	1.2	0.69	-3.67	-2.47

Table S13. BCP properties of **2_Co**

A - B		d1	d2	f	del2f	ell	Gb	Gb/f	Vb	Eb
CO(1)	-N(4)	1.0028	1.021	0.57	8.72	0.06	0.72	1.27	-0.83	-0.11
CO(1)	-N(1)	1.0032	1.0167	0.58	8.85	0.07	0.73	1.27	-0.85	-0.11
CO(1)	-N(2)	1.0053	1.0233	0.56	8.62	0.08	0.71	1.26	-0.82	-0.11
CO(1)	-N(3)	1.005	1.0235	0.56	8.62	0.08	0.71	1.26	-0.81	-0.11
S(1)	-N(1)	0.7693	0.8473	1.72	-9.2	0.1	1.55	0.9	-3.74	-2.19
S(1)	-N(2)	0.7735	0.8478	1.71	-8.9	0.1	1.54	0.9	-3.71	-2.16
C(1)	-S(1)	0.896	0.9025	1.3	-5.34	0	1	0.77	-2.37	-1.37
S(2)	-N(3)	0.7756	0.848	1.7	-8.76	0.1	1.54	0.9	-3.69	-2.15
S(2)	-N(4)	0.7653	0.847	1.73	-9.48	0.1	1.56	0.9	-3.78	-2.22
C(13)	-S(2)	0.8954	0.9016	1.3	-5.39	0.03	1	0.77	-2.38	-1.38
SI(1)	-N(1)	0.7293	1.0025	0.98	4.93	0.01	1	1.03	-1.66	-0.66
SI(1)	-C(7)	0.7685	1.0905	0.91	-0.64	0.01	0.65	0.72	-1.35	-0.7
SI(1)	-C(8)	0.7697	1.0937	0.9	-0.7	0.01	0.65	0.72	-1.34	-0.7

SI(1)	-C(9)	0.77	1.094	0.9	-0.69	0	0.65	0.71	-1.34	-0.69
SI(2)	-N(2)	0.7306	1.0055	0.97	4.74	0.01	0.99	1.02	-1.65	-0.66
SI(2)	-C(10)	0.768	1.09	0.91	-0.61	0.01	0.66	0.72	-1.36	-0.7
SI(2)	-C(11)	0.7682	1.0903	0.91	-0.6	0	0.66	0.72	-1.36	-0.7
SI(2)	-C(12)	0.7673	1.0896	0.91	-0.56	0.01	0.66	0.73	-1.36	-0.7
SI(3)	-N(3)	0.7312	1.0065	0.97	4.66	0.01	0.98	1.01	-1.64	-0.66
SI(3)	-C(19)	0.7723	1.0971	0.9	-0.83	0	0.63	0.7	-1.32	-0.69
SI(3)	-C(20)	0.771	1.0956	0.9	-0.77	0	0.64	0.71	-1.33	-0.69
SI(3)	-C(21)	0.771	1.095	0.9	-0.78	0.01	0.64	0.71	-1.33	-0.69
SI(4)	-N(4)	0.7299	1.0034	0.98	4.86	0.01	1	1.02	-1.66	-0.66
SI(4)	-C(22)	0.7687	1.0912	0.91	-0.65	0.01	0.65	0.72	-1.35	-0.7
SI(4)	-C(23)	0.7668	1.0887	0.91	-0.53	0.01	0.67	0.73	-1.37	-0.7
SI(4)	-C(24)	0.7671	1.0881	0.91	-0.55	0.01	0.66	0.73	-1.37	-0.7
C(2)	-C(1)	0.6895	0.7008	2.19	-19.96	0.19	2.04	0.93	-5.49	-3.44
C(6)	-C(1)	0.6883	0.7004	2.21	-20.26	0.19	2.06	0.93	-5.53	-3.48
H(2)	-C(2)	0.3407	0.7423	1.77	-18.04	0.06	1.24	0.7	-3.74	-2.5
H(4)	-C(4)	0.3577	0.7253	1.75	-16.79	0.03	1.27	0.72	-3.71	-2.44
H(6)	-C(6)	0.3408	0.7422	1.77	-18.02	0.06	1.23	0.7	-3.73	-2.5
C(3)	-C(2)	0.6946	0.6967	2.17	-19.78	0.18	2.01	0.92	-5.39	-3.39
H(3)	-C(3)	0.3617	0.7214	1.74	-16.78	0.09	1.23	0.71	-3.64	-2.41
H(5)	-C(5)	0.3618	0.7212	1.73	-16.77	0.09	1.23	0.71	-3.64	-2.4
C(3)	-C(4)	0.6831	0.7128	2.24	-22.2	0.2	2.04	0.91	-5.63	-3.59
C(5)	-C(4)	0.6815	0.7115	2.25	-22.42	0.19	2.05	0.91	-5.67	-3.62
C(5)	-C(6)	0.6984	0.7009	2.15	-19.17	0.18	1.98	0.92	-5.3	-3.32
H(7A)	-C(7)	0.4095	0.6682	1.67	-15.15	0.08	1.19	0.71	-3.44	-2.25
H(7B)	-C(7)	0.4098	0.6679	1.67	-15.16	0.08	1.19	0.71	-3.44	-2.25
H(7C)	-C(7)	0.4099	0.6681	1.67	-15.03	0.08	1.19	0.71	-3.42	-2.24
H(8A)	-C(8)	0.4102	0.6678	1.67	-15.04	0.08	1.18	0.71	-3.42	-2.24
H(8B)	-C(8)	0.4104	0.6679	1.66	-14.85	0.07	1.18	0.71	-3.4	-2.22
H(8C)	-C(8)	0.4101	0.6679	1.67	-14.98	0.07	1.18	0.71	-3.42	-2.23
H(9A)	-C(9)	0.4105	0.6676	1.66	-14.94	0.07	1.18	0.71	-3.41	-2.23
H(9B)	-C(9)	0.4102	0.668	1.66	-14.92	0.07	1.18	0.71	-3.41	-2.23
H(9C)	-C(9)	0.4104	0.6679	1.66	-14.87	0.07	1.18	0.71	-3.4	-2.22
H(10A)	-C(10)	0.41	0.6681	1.67	-15	0.07	1.19	0.71	-3.42	-2.24
H(10B)	-C(10)	0.41	0.6677	1.67	-15.17	0.08	1.19	0.71	-3.43	-2.25
H(10C)	-C(10)	0.4099	0.6682	1.67	-14.95	0.07	1.19	0.71	-3.42	-2.23
H(11A)	-C(11)	0.41	0.6683	1.67	-14.87	0.07	1.19	0.71	-3.41	-2.23
H(11B)	-C(11)	0.4103	0.6679	1.67	-14.97	0.07	1.18	0.71	-3.41	-2.23
H(11C)	-C(11)	0.4099	0.6677	1.67	-15.16	0.08	1.19	0.71	-3.43	-2.25
H(12A)	-C(12)	0.4103	0.6677	1.67	-15	0.07	1.18	0.71	-3.42	-2.23
H(12B)	-C(12)	0.41	0.6679	1.67	-15.07	0.08	1.19	0.71	-3.43	-2.24
H(12C)	-C(12)	0.4106	0.6681	1.66	-14.61	0.06	1.18	0.71	-3.38	-2.2
C(14)	-C(13)	0.6896	0.7005	2.19	-19.95	0.19	2.05	0.93	-5.49	-3.44
C(18)	-C(13)	0.6894	0.7015	2.2	-20.11	0.19	2.05	0.93	-5.51	-3.46
H(14)	-C(14)	0.3407	0.7423	1.77	-18.03	0.06	1.24	0.7	-3.74	-2.5
H(16)	-C(16)	0.3577	0.7254	1.75	-16.78	0.03	1.27	0.72	-3.71	-2.44
H(18)	-C(18)	0.3408	0.7423	1.77	-18.05	0.06	1.23	0.7	-3.73	-2.5

C(15)	-C(14)	0.6946	0.6966	2.17	-19.82	0.18	2	0.92	-5.4	-3.39
H(15)	-C(15)	0.3616	0.7214	1.74	-16.79	0.09	1.23	0.71	-3.64	-2.41
H(17)	-C(17)	0.3617	0.7213	1.74	-16.79	0.09	1.23	0.71	-3.64	-2.41
C(15)	-C(16)	0.6806	0.7103	2.25	-22.62	0.2	2.06	0.91	-5.7	-3.64
C(17)	-C(16)	0.6812	0.7114	2.25	-22.42	0.19	2.05	0.91	-5.68	-3.62
C(17)	-C(18)	0.6959	0.6984	2.16	-19.54	0.18	2	0.92	-5.36	-3.36
H(19A)	-C(19)	0.4099	0.6682	1.67	-15.03	0.08	1.19	0.71	-3.43	-2.24
H(19B)	-C(19)	0.4099	0.6678	1.67	-15.15	0.08	1.19	0.71	-3.43	-2.25
H(19C)	-C(19)	0.4097	0.668	1.67	-15.13	0.08	1.19	0.71	-3.44	-2.25
H(20A)	-C(20)	0.4099	0.6677	1.67	-15.19	0.09	1.19	0.71	-3.44	-2.25
H(20B)	-C(20)	0.4102	0.668	1.66	-14.92	0.07	1.18	0.71	-3.41	-2.23
H(20C)	-C(20)	0.4098	0.6682	1.67	-15	0.08	1.19	0.71	-3.43	-2.24
H(21A)	-C(21)	0.4097	0.6679	1.67	-15.18	0.09	1.19	0.71	-3.44	-2.25
H(21B)	-C(21)	0.4099	0.668	1.67	-15.07	0.08	1.19	0.71	-3.43	-2.24
H(21C)	-C(21)	0.4097	0.6682	1.67	-15.1	0.08	1.19	0.71	-3.44	-2.25
H(22A)	-C(22)	0.4097	0.6681	1.67	-15.13	0.08	1.19	0.71	-3.44	-2.25
H(22B)	-C(22)	0.4099	0.668	1.67	-15.06	0.08	1.19	0.71	-3.43	-2.24
H(22C)	-C(22)	0.4101	0.6679	1.67	-15.03	0.08	1.18	0.71	-3.42	-2.24
H(23A)	-C(23)	0.4101	0.6681	1.66	-14.89	0.07	1.18	0.71	-3.41	-2.23
H(23B)	-C(23)	0.4105	0.6678	1.66	-14.87	0.07	1.18	0.71	-3.4	-2.22
H(23C)	-C(23)	0.4105	0.6677	1.66	-14.9	0.07	1.18	0.71	-3.41	-2.22
H(24A)	-C(24)	0.4102	0.6678	1.67	-15.02	0.07	1.18	0.71	-3.42	-2.24
H(24B)	-C(24)	0.4098	0.6683	1.67	-14.98	0.08	1.19	0.71	-3.42	-2.24
H(24C)	-C(24)	0.4097	0.668	1.67	-15.16	0.09	1.19	0.71	-3.44	-2.25

Table S14. Bader charges of 1_Co

Atom	q				
CO(1)	0.9223	C(13)	0.0717	H(6)	0.1694
S(1)	0.9672	C(14)	0.0802	H(8A)	-0.0246
S(2)	0.9718	C(15)	-0.156	H(8B)	-0.0269
N(1)	-1.1391	C(16)	-0.1705	H(8C)	-0.0216
N(2)	-1.147	C(17)	-0.1301	H(9A)	-0.0217
N(3)	-1.1372	C(18)	-0.1373	H(9B)	-0.022
N(4)	-1.1361	C(19)	-0.1268	H(9C)	-0.0223
C(1)	-0.1626	C(20)	-0.1708	H(10A)	-0.0211
C(2)	-0.1718	C(21)	0.4896	H(10B)	-0.0226
C(3)	-0.1264	C(22)	0.076	H(10C)	-0.0212
C(4)	-0.182	C(23)	0.0819	H(12A)	-0.021
C(5)	-0.1289	C(24)	0.0877	H(12B)	-0.0277
C(6)	-0.1715	C(25)	0.472	H(12C)	-0.0238
C(7)	0.5054	C(26)	0.0802	H(13A)	-0.024
C(8)	0.0749	C(27)	0.082	H(13B)	-0.022
C(9)	0.0758	C(28)	0.0743	H(13C)	-0.025
C(10)	0.0694	H(2)	0.1679	H(14A)	-0.0221
C(11)	0.4962	H(3)	0.1537	H(14B)	-0.0255
C(12)	0.0724	H(4)	0.1629	H(14C)	-0.023
		H(5)	0.1537	H(16)	0.1668

H(17)	0.1538	H(23B)	-0.0235	H(27A)	-0.0218
H(18)	0.114	H(23C)	-0.0219	H(27B)	-0.0221
H(19)	0.1532	H(24A)	-0.024	H(27C)	-0.0248
H(20)	0.1685	H(24B)	-0.0246	H(28A)	-0.0224
H(22A)	-0.0286	H(24C)	-0.0225	H(28B)	-0.0237
H(22B)	-0.0243	H(26A)	-0.0214	H(28C)	-0.0208
H(22C)	-0.0216	H(26B)	-0.0255		
H(23A)	-0.0241	H(26C)	-0.0261		

Table S15. Bader charges of 2_Co

Atom	q	C(17)	-0.1078	H(12A)	0.0393
CO(1)	0.8638	C(18)	-0.2623	H(12B)	0.0391
S(1)	0.9654	C(19)	-0.6355	H(12C)	0.047
S(2)	0.9505	C(20)	-0.6198	H(14)	0.1988
SI(1)	2.4539	C(21)	-0.6025	H(15)	0.1637
SI(2)	2.4492	C(22)	-0.6412	H(16)	0.1159
SI(3)	2.473	C(23)	-0.629	H(17)	0.1636
SI(4)	2.4766	C(24)	-0.6242	H(18)	0.2004
N(1)	-1.4526	H(2)	0.1988	H(19A)	0.0402
N(2)	-1.4531	H(3)	0.1637	H(19B)	0.0371
N(3)	-1.4483	H(4)	0.1156	H(19C)	0.0376
N(4)	-1.456	H(5)	0.1638	H(20A)	0.037
C(1)	-0.2886	H(6)	0.2014	H(20B)	0.0418
C(2)	-0.2588	H(7A)	0.0376	H(20C)	0.041
C(3)	-0.1155	H(7B)	0.0371	H(21A)	0.0367
C(4)	-0.1382	H(7C)	0.0393	H(21B)	0.0405
C(5)	-0.1091	H(8A)	0.039	H(21C)	0.0384
C(6)	-0.2623	H(8B)	0.0412	H(22A)	0.0377
C(7)	-0.6141	H(8C)	0.0404	H(22B)	0.0399
C(8)	-0.6324	H(9A)	0.0403	H(22C)	0.0396
C(9)	-0.6374	H(9B)	0.0414	H(23A)	0.0425
C(10)	-0.6252	H(9C)	0.0423	H(23B)	0.0423
C(11)	-0.6275	H(10A)	0.0409	H(23C)	0.0407
C(12)	-0.6307	H(10B)	0.0372	H(24A)	0.0385
C(13)	-0.2909	H(10C)	0.0408	H(24B)	0.041
C(14)	-0.2542	H(11A)	0.0426	H(24C)	0.0365
C(15)	-0.1083	H(11B)	0.0405		
C(16)	-0.142	H(11C)	0.0369		

Theoretical calculations

```
! def2-TZVP def2-TZVP/C PATOM tightscf Keepdens
! SlowConv KDIIS SOSCF
%maxcore 5000
%pal
nprocs 36
end
%casscf nel 7
        norb 5 #7 electrons in 5 d orbitals
        mult 4,2 # 10 quartet and 40 doublet states
        nroots 10,40
        trafostep RI
        #-----------------------------------------------
        nevpt2 SC #Perform the SC-NEVPT2 correction
        actorbs dorbs    #Makes the ab initio LFT analysis
        #-----------------------------------------------
        rel #flag for relativistic properties
                printlevel 3            #Control the amount of printing
                dosoc true              #Do the SOC calculation
                nroots 4
                #-----------------------------------------------
                mcd true   # Request the MCD calculation
                NInitStates 28          # Number of Donor SOC states
                                        # for the ABS and MCD spectra evaluation
                NPointsTheta 10 # Number of integration point for
                NPointsPhi 10           # Euler angles
                NPointsPsi 10           #
                B 5000                  # Experimental Magnetic field (in Gauss)
                Temperature 10          # Experimental temperature (in K)
                #-----------------------------------------------
                domagnetization true
                dosusceptibility true
                LebedevPrec 5   # Precision of the grid for different field
                                        # directions (meaningful values range from 1
                                        # (smallest) to 10 (largest))
                nPointsFStep 5 # number of steps for numerical differentiation
                                        # (def: 5, meaningful values are 3, 5 7 and 9)
                MAGFieldStep 100.0 # Size of field step for numerical differentiation
                                        # (def: 100 Gauss)
                MAGTemperatureMIN 4.0 # minimum temperature (K) for magnetization
                MAGTemperatureMAX 4.0 # maximum temperature (K) for magnetization
                MAGTemperatureNPoints 1 # number of temperature points for magnetization
                MAGFieldMIN 0.0 # minimum field (Gauss) for magnetization
                MAGFieldMAX 70000.0 # maximum field (Gauss) for magnetization
                MAGNpoints 15 # number of field points for magnetization
                SUSTempMIN 1.0 # minimum temperature (K) for susceptibility
                SUSTempMAX 300.0 # maximum temperature (K) for susceptibility
                SUSNPoints 300 # number of temperature points for susceptibility
                SUSStatFieldMIN 0.0 # minimum static field (Gauss) for susceptibility
                SUSStatFieldMAX 0.1 # maximum static field (Gauss) for susceptibility
                SUSStatFieldNPoints 2 # number of static fields for susceptibility
                #-----------------------------------------------
                gtensor true            # Request the G-tensor Calculation
                #-----------------------------------------------
                dtensor true            # Request the ZFS-tensor Calculation
                                        #(default if dosoc true)
                #-----------------------------------------------
        end
end
!Printbasis
%scf print[p_mos] 1
end
* xyzfile 0  4 M1.xyz
```

Table S16. xyz for **1_Co**.

CO(1)	0	0	0

CO(1B)	0	0	0
S(1)	0.007896	0.006279	-0.13952
S(1B)	0.007896	0.006279	-0.13952
S(2)	0.007567	-0.00769	0.139446
S(2B)	0.007567	-0.00769	0.139446
N(1)	-0.038379	-0.037241	-0.090146
N(1B)	-0.038379	-0.037241	-0.090146
N(2)	0.044269	0.049252	-0.082022
N(2B)	0.044269	0.049252	-0.082022
N(3)	0.039377	-0.053846	0.081714
N(3B)	0.039377	-0.053846	0.081714
N(4)	-0.034328	0.040351	0.090341
N(4B)	-0.034328	0.040351	0.090341
C(1)	-0.046911	0.062591	-0.182911
C(1B)	-0.046911	0.062591	-0.182911
C(2)	-0.09341	0.102152	-0.149386
C(2B)	-0.09341	0.102152	-0.149386
C(3)	-0.134636	0.144705	-0.185342
C(3B)	-0.134636	0.144705	-0.185342
C(4)	-0.129734	0.14793	-0.254779
C(4B)	-0.129734	0.14793	-0.254779
C(5)	-0.083459	0.108411	-0.288137
C(5B)	-0.083459	0.108411	-0.288137
C(6)	-0.042011	0.065675	-0.252057
C(6B)	-0.042011	0.065675	-0.252057
C(7)	-0.069932	-0.099257	-0.113764
C(7B)	-0.069932	-0.099257	-0.113764
C(8)	-0.103992	-0.129705	-0.052449
C(8B)	-0.103992	-0.129705	-0.052449
C(9)	-0.123524	-0.086558	-0.1672
C(9B)	-0.123524	-0.086558	-0.1672
C(10)	-0.01861	-0.149488	-0.140955
C(10B)	-0.01861	-0.149488	-0.140955
C(11)	0.10783	0.083314	-0.096297
C(11B)	0.10783	0.083314	-0.096297
C(12)	0.12522	0.122381	-0.03291
C(12B)	0.12522	0.122381	-0.03291
C(13)	0.164232	0.033405	-0.111245
C(13B)	0.164232	0.033405	-0.111245
C(14)	0.102557	0.133177	-0.154375
C(14B)	0.102557	0.133177	-0.154375
C(15)	-0.052405	-0.058448	0.182849
C(15B)	-0.052405	-0.058448	0.182849
C(16)	-0.102768	-0.093	0.149367
C(16B)	-0.102768	-0.093	0.149367
C(17)	-0.147859	-0.131434	0.185324
C(17B)	-0.147859	-0.131434	0.185324

C(18)	-0.142986	-0.135498	0.254721
C(18B)	-0.142986	-0.135498	0.254721
C(19)	-0.09287	-0.100942	0.288036
C(19B)	-0.09287	-0.100942	0.288036
C(20)	-0.04754	-0.062349	0.251955
C(20B)	-0.04754	-0.062349	0.251955
C(21)	0.099352	-0.093993	0.095675
C(21B)	0.099352	-0.093993	0.095675
C(22)	0.112624	-0.13441	0.03214
C(22B)	0.112624	-0.13441	0.03214
C(23)	0.089436	-0.143256	0.153656
C(23B)	0.089436	-0.143256	0.153656
C(24)	0.160408	-0.049869	0.110526
C(24B)	0.160408	-0.049869	0.110526
C(25)	-0.059435	0.105154	0.114229
C(25B)	-0.059435	0.105154	0.114229
C(26)	-0.090588	0.138976	0.053137
C(26B)	-0.090588	0.138976	0.053137
C(27)	-0.113763	0.097703	0.167912
C(27B)	-0.113763	0.097703	0.167912
C(28)	-0.003246	0.149974	0.14125
C(28B)	-0.003246	0.149974	0.14125
H(2)	-0.097178	0.099415	-0.096045
H(3)	-0.170492	0.175243	-0.159557
H(4)	-0.161787	0.180955	-0.282432
H(5)	-0.079526	0.110694	-0.341629
H(6)	-0.006133	0.034992	-0.277705
H(8A)	-0.140732	-0.095327	-0.032232
H(8B)	-0.067694	-0.14083	-0.013801
H(8C)	-0.129336	-0.175828	-0.065521
H(9A)	-0.14969	-0.132597	-0.179029
H(9B)	-0.101999	-0.067881	-0.213272
H(9C)	-0.159782	-0.050288	-0.149754
H(10A)	-0.042854	-0.196105	-0.154499
H(10B)	0.006345	-0.130583	-0.185189
H(10C)	0.01919	-0.160215	-0.103666
H(12A)	0.172846	0.147722	-0.038489
H(12B)	0.12875	0.089426	0.009941
H(12C)	0.087249	0.1596	-0.022562
H(13A)	0.15314	0.003677	-0.155196
H(13B)	0.211082	0.059285	-0.120223
H(13C)	0.171429	-0.000382	-0.069519
H(14A)	0.148654	0.161368	-0.159153
H(14B)	0.09404	0.108116	-0.201676
H(14C)	0.061967	0.168101	-0.146251
H(16)	-0.106496	-0.089582	0.096066
H(17)	-0.186695	-0.158118	0.159578

H(18)	-0.178035	-0.165325	0.282373
H(19)	-0.088954	-0.103871	0.341498
H(20)	-0.00869	-0.0355	0.277575
H(22A)	0.119202	-0.101857	-0.010656
H(22B)	0.071158	-0.167708	0.021855
H(22C)	0.157563	-0.164298	0.037494
H(23A)	0.08363	-0.117615	0.201056
H(23B)	0.045572	-0.173993	0.14563
H(23C)	0.132554	-0.175862	0.158162
H(24A)	0.152424	-0.019306	0.154582
H(24B)	0.170717	-0.016836	0.068848
H(24C)	0.204543	-0.08021	0.119286
H(26A)	-0.111177	0.187353	0.06642
H(26B)	-0.13064	0.108465	0.033008
H(26C)	-0.053533	0.146564	0.014345
H(27A)	-0.153506	0.065229	0.150586
H(27B)	-0.135198	0.146073	0.179953
H(27C)	-0.093961	0.076891	0.213843
H(28A)	-0.022702	0.198713	0.155041
H(28B)	0.019957	0.128568	0.185296
H(28C)	0.035234	0.157037	0.10378

Table S17. xyz for **2_Co**.

CO(1)	0	0	0
CO(1B)	0	0	0
S(1)	0.010857	0.000086	0.137331
S(1B)	0.010857	0.000086	0.137331
S(2)	0.010865	-0.000116	-0.13731
S(2B)	0.010865	-0.000116	-0.13731
SI(1)	0.111773	0.100276	0.095721
SI(1B)	0.111773	0.100276	0.095721
SI(2)	-0.073698	-0.117878	0.112878
SI(2B)	-0.073698	-0.117878	0.112878
SI(3)	-0.073414	0.11806	-0.112801
SI(3B)	-0.073414	0.11806	-0.112801
SI(4)	0.111418	-0.100697	-0.095732
SI(4B)	0.111418	-0.100697	-0.095732
N(1)	0.044829	0.047345	0.08276
N(1B)	0.044829	0.047345	0.08276
N(2)	-0.035805	-0.043806	0.089488
N(2B)	-0.035805	-0.043806	0.089488
N(3)	-0.03567	0.043903	-0.089471
N(3B)	-0.03567	0.043903	-0.089471
N(4)	0.044709	-0.047468	-0.082738
N(4B)	0.044709	-0.047468	-0.082738
C(1)	-0.044041	0.052145	0.18512

C(1B)	-0.044041	0.052145	0.18512
C(2)	-0.096458	0.08781	0.156298
C(2B)	-0.096458	0.08781	0.156298
C(3)	-0.136629	0.12782	0.196096
C(3B)	-0.136629	0.12782	0.196096
C(4)	-0.124746	0.132298	0.26462
C(4B)	-0.124746	0.132298	0.26462
C(5)	-0.072636	0.096576	0.293257
C(5B)	-0.072636	0.096576	0.293257
C(6)	-0.03219	0.056312	0.253349
C(6B)	-0.03219	0.056312	0.253349
C(7)	0.151874	0.114854	0.011964
C(7B)	0.151874	0.114854	0.011964
C(8)	0.084599	0.183532	0.130446
C(8B)	0.084599	0.183532	0.130446
C(9)	0.175074	0.063051	0.154845
C(9B)	0.175074	0.063051	0.154845
C(10)	-0.11088	-0.155097	0.035009
C(10B)	-0.11088	-0.155097	0.035009
C(11)	-0.142965	-0.104564	0.175353
C(11B)	-0.142965	-0.104564	0.175353
C(12)	-0.012235	-0.178732	0.15034
C(12B)	-0.012235	-0.178732	0.15034
C(13)	-0.044183	-0.052025	-0.185093
C(13B)	-0.044183	-0.052025	-0.185093
C(14)	-0.096669	-0.087569	-0.156242
C(14B)	-0.096669	-0.087569	-0.156242
C(15)	-0.13695	-0.127489	-0.196018
C(15B)	-0.13695	-0.127489	-0.196018
C(16)	-0.12511	-0.131997	-0.264548
C(16B)	-0.12511	-0.131997	-0.264548
C(17)	-0.072934	-0.096393	-0.293212
C(17B)	-0.072934	-0.096393	-0.293212
C(18)	-0.032376	-0.056219	-0.253326
C(18B)	-0.032376	-0.056219	-0.253326
C(19)	-0.14277	0.104907	-0.175209
C(19B)	-0.14277	0.104907	-0.175209
C(20)	-0.011862	0.178781	-0.150328
C(20B)	-0.011862	0.178781	-0.150328
C(21)	-0.110438	0.155367	-0.034911
C(21B)	-0.110438	0.155367	-0.034911
C(22)	0.083835	-0.183978	-0.130055
C(22B)	0.083835	-0.183978	-0.130055
C(23)	0.174636	-0.063908	-0.155219
C(23B)	0.174636	-0.063908	-0.155219
C(24)	0.151755	-0.115103	-0.012059
C(24B)	0.151755	-0.115103	-0.012059

H(2)	-0.10565	0.084059	0.103675
H(3)	-0.177028	0.155426	0.17401
H(4)	-0.156034	0.163356	0.295254
H(5)	-0.063431	0.099784	0.346041
H(6)	0.008134	0.028393	0.275249
H(7A)	0.192959	0.150122	0.016402
H(7B)	0.116454	0.135447	-0.023651
H(7C)	0.171715	0.068931	-0.009048
H(8A)	0.127149	0.21689	0.136034
H(8B)	0.061181	0.178423	0.179151
H(8C)	0.04882	0.207709	0.097517
H(9A)	0.19266	0.014769	0.137321
H(9B)	0.154631	0.05603	0.204668
H(9C)	0.218188	0.095801	0.159487
H(10A)	-0.073123	-0.164611	-0.00279
H(10B)	-0.148426	-0.12218	0.013638
H(10C)	-0.135133	-0.202368	0.046345
H(11A)	-0.124492	-0.08468	0.222372
H(11B)	-0.167984	-0.151579	0.186223
H(11C)	-0.180196	-0.069887	0.156409
H(12A)	0.027289	-0.191169	0.115265
H(12B)	-0.037142	-0.224965	0.164315
H(12C)	0.011185	-0.158664	0.195037
H(14)	-0.105829	-0.083788	-0.103616
H(15)	-0.177401	-0.155006	-0.173916
H(16)	-0.156483	-0.162988	-0.295163
H(17)	-0.06376	-0.099621	-0.346
H(18)	0.007999	-0.028396	-0.275252
H(19A)	-0.167768	0.151953	-0.185982
H(19B)	-0.18001	0.070231	-0.156278
H(19C)	-0.12436	0.085078	-0.222277
H(20A)	0.027732	0.191145	-0.115307
H(20B)	-0.036686	0.22506	-0.164295
H(20C)	0.011469	0.158647	-0.195044
H(21A)	-0.072623	0.164818	0.002845
H(21B)	-0.148023	0.12252	-0.013498
H(21C)	-0.134629	0.202675	-0.046226
H(22A)	0.060175	-0.17895	-0.178653
H(22B)	0.048158	-0.207961	-0.096872
H(22C)	0.126255	-0.21748	-0.135763
H(23A)	0.217665	-0.096774	-0.159817
H(23B)	0.192382	-0.015579	-0.137987
H(23C)	0.154068	-0.057096	-0.205019
H(24A)	0.192591	-0.150662	-0.016473
H(24B)	0.116351	-0.135268	0.023817
H(24C)	0.171978	-0.069193	0.008615

Multipole refinement against theoretical structure factors

Table S18. refinement steps of the multipole refinement against theoretical structure factors.

Step	Refined parameters
1	Monopoles
2	+ Multipoles up to hexadecapoles (H: quadrupoles)
3	+ κ of non-hydrogen atoms
4	+ κ of core electrons of non-hydrogen atoms
5	κ' of non-hydrogen atoms
6	as step 4
7	+ quadrupoles at hydrogen atoms

Table S19. Crystal data and structure refinement for **1_Co** and **2_Co**.

	**1_Co**	**2_Co**
a (Å)	20.000	20.000
β (°)	90.000	90.000
Z	1	1
F(000)	301	333
**Multipole Refinement**		
Data / parameters	4163.1745	4808.6279
$R_1(F^2)$	0.0032	0.0029
GOF	0.0227	0.0232
$\Delta\rho_{max}$, $\Delta\rho_{min}$ (e Å^{-3})	0.370, -0.282	0.464, -0.270

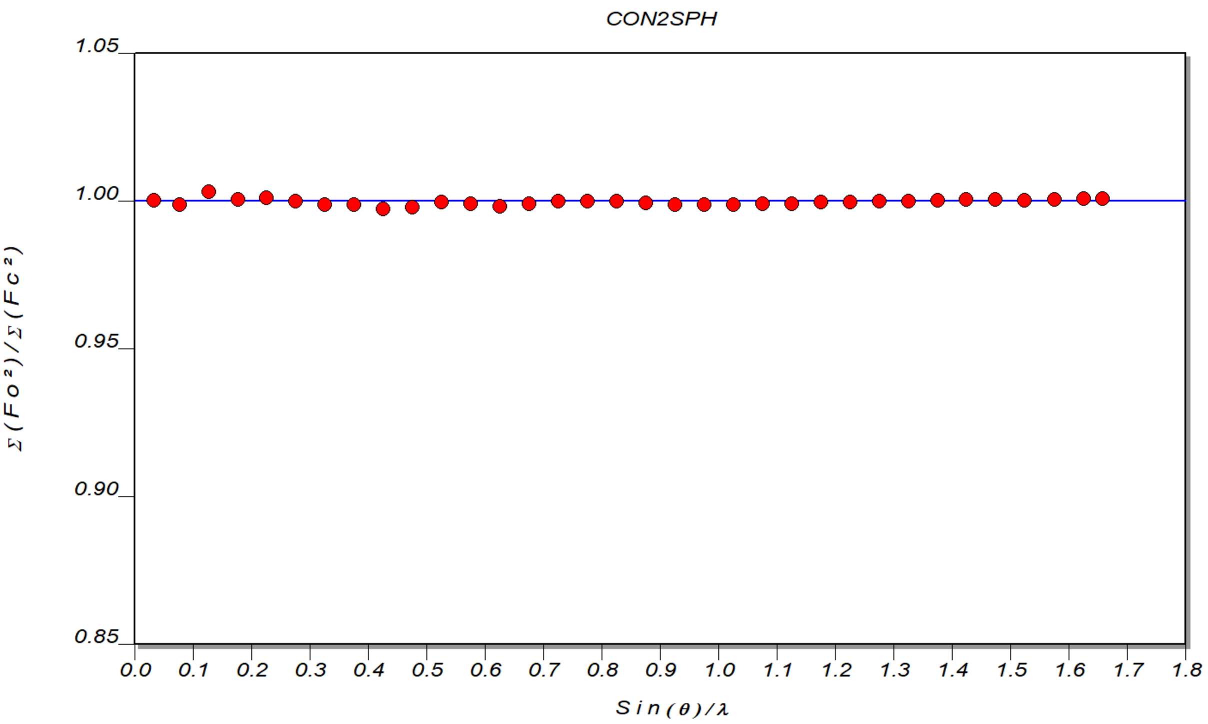

Figure S15. DRK plot[11] **of 1_Co**.

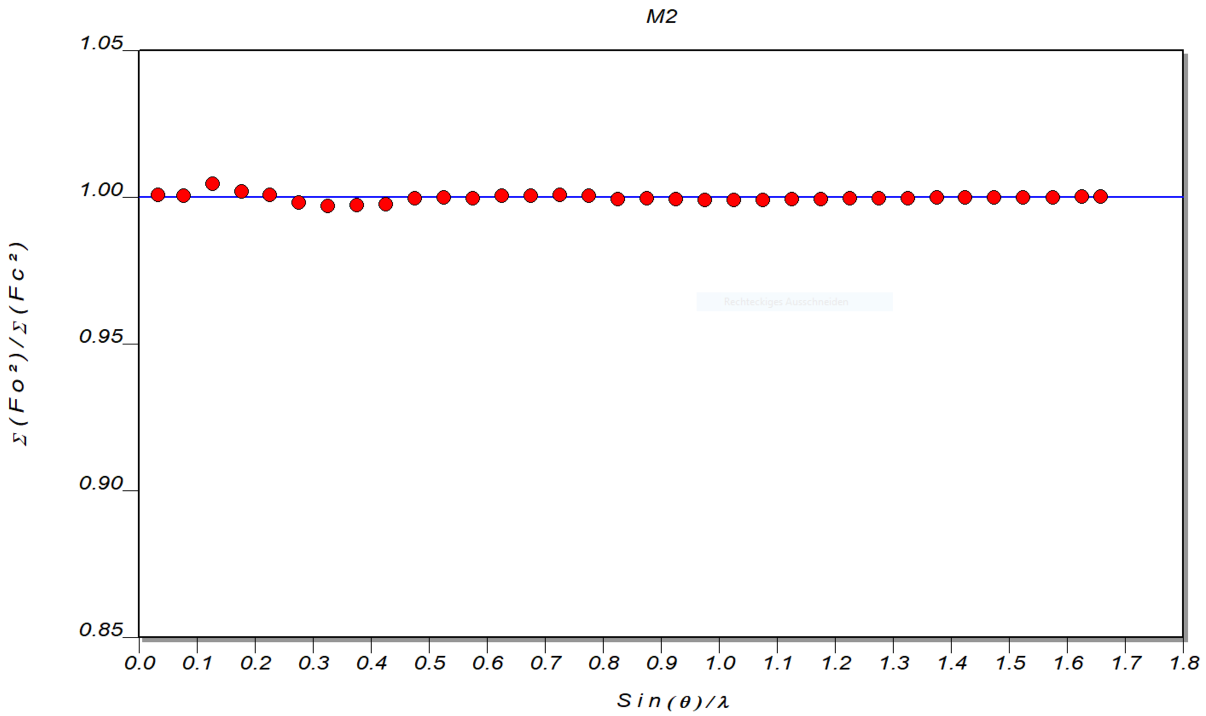

Figure S16. DRK plot[11] **of 2_Co**.

Table S20. BCP properties of **1_Co**

A - B	d1	d2	f	del2f	ell	Gb	Gb/f	Vb	Eb
CO(1) -N(1)	1.0298	1.0673	0.48	7.06	0.03	0.57	1.18	-0.64	-0.07
CO(1) -N(2)	1.0273	1.0817	0.44	7.34	0.02	0.55	1.24	-0.58	-0.03
CO(1) -N(3)	1.0267	1.0832	0.44	7.31	0.06	0.54	1.24	-0.58	-0.03
CO(1) -N(4)	1.0284	1.0669	0.48	7.11	0.01	0.57	1.18	-0.64	-0.07
S(1) -N(1)	0.781	0.8292	1.79	-13.25	0.16	1.5	0.84	-3.92	-2.42
S(1) -N(2)	0.7815	0.8289	1.79	-13.21	0.16	1.5	0.84	-3.92	-2.42
C(1) -S(1)	0.8603	0.935	1.36	-6.99	0.03	1.02	0.75	-2.53	-1.51
C(7) -N(1)	0.6066	0.8631	1.74	-15.88	0.04	1.28	0.74	-3.67	-2.39
C(11) -N(2)	0.6071	0.8633	1.74	-15.88	0.04	1.28	0.73	-3.66	-2.39
S(2) -N(3)	0.7814	0.8289	1.79	-13.22	0.16	1.5	0.84	-3.92	-2.42
S(2) -N(4)	0.7812	0.8291	1.79	-13.23	0.16	1.5	0.84	-3.92	-2.42
C(15) -S(2)	0.8603	0.9349	1.36	-6.99	0.03	1.02	0.75	-2.53	-1.51
C(21) -N(3)	0.607	0.8633	1.74	-15.88	0.04	1.28	0.73	-3.66	-2.39
C(25) -N(4)	0.6067	0.8631	1.74	-15.88	0.04	1.28	0.74	-3.67	-2.39
C(1) -C(2)	0.6941	0.6991	2.16	-20.52	0.22	1.95	0.9	-5.34	-3.39
C(1) -C(6)	0.6921	0.6956	2.18	-21.05	0.22	1.97	0.9	-5.42	-3.45
H(2) -C(2)	0.3755	0.6954	2	-24.12	0.03	1.42	0.71	-4.54	-3.11
H(4) -C(4)	0.3769	0.697	2	-24.28	0.03	1.41	0.71	-4.52	-3.11
H(6) -C(6)	0.3768	0.6978	1.99	-23.8	0.03	1.41	0.71	-4.49	-3.08
C(3) -C(2)	0.6911	0.695	2.2	-21.66	0.21	1.97	0.9	-5.46	-3.49
H(3) -C(3)	0.3782	0.6957	1.99	-23.97	0.04	1.41	0.71	-4.49	-3.08
H(5) -C(5)	0.3782	0.6955	1.99	-23.98	0.04	1.41	0.71	-4.49	-3.08
C(3) -C(4)	0.6935	0.7002	2.17	-20.75	0.21	1.96	0.9	-5.37	-3.41

C(5)	-C(4)	0.6906	0.6974	2.19	-21.23	0.21	1.98	0.9	-5.44	-3.46
C(5)	-C(6)	0.6941	0.6982	2.18	-21.13	0.21	1.95	0.9	-5.39	-3.43
C(7)	-C(8)	0.7631	0.7665	1.72	-16.48	0.03	1.23	0.71	-3.6	-2.38
C(7)	-C(9)	0.7653	0.7697	1.71	-16.23	0.03	1.21	0.71	-3.56	-2.35
C(7)	-C(10)	0.7659	0.7702	1.71	-16.12	0.03	1.21	0.71	-3.54	-2.34
H(8A)	-C(8)	0.4185	0.666	1.89	-22.79	0.01	1.25	0.66	-4.1	-2.85
H(8B)	-C(8)	0.4181	0.6654	1.89	-22.85	0.01	1.25	0.66	-4.11	-2.85
H(8C)	-C(8)	0.418	0.6665	1.89	-22.77	0.01	1.26	0.67	-4.11	-2.85
H(9A)	-C(9)	0.4186	0.6666	1.88	-22.74	0.01	1.25	0.66	-4.09	-2.84
H(9B)	-C(9)	0.4181	0.6655	1.89	-22.83	0.01	1.25	0.66	-4.11	-2.85
H(9C)	-C(9)	0.418	0.6655	1.89	-22.86	0.01	1.26	0.66	-4.11	-2.86
H(10A)	-C(10)	0.4187	0.6666	1.88	-22.74	0.01	1.25	0.66	-4.09	-2.84
H(10B)	-C(10)	0.418	0.6659	1.89	-22.8	0.01	1.26	0.67	-4.11	-2.85
H(10C)	-C(10)	0.4182	0.6652	1.89	-22.87	0.01	1.25	0.66	-4.11	-2.85
C(11)	-C(12)	0.763	0.7665	1.73	-16.51	0.03	1.23	0.71	-3.61	-2.38
C(11)	-C(13)	0.7658	0.77	1.71	-16.2	0.03	1.21	0.71	-3.55	-2.34
C(11)	-C(14)	0.7652	0.7696	1.71	-16.2	0.03	1.21	0.71	-3.55	-2.34
H(12A)	-C(12)	0.4181	0.6667	1.89	-22.76	0.01	1.26	0.67	-4.1	-2.85
H(12B)	-C(12)	0.4182	0.6653	1.89	-22.85	0.01	1.25	0.66	-4.11	-2.85
H(12C)	-C(12)	0.4181	0.6653	1.89	-22.87	0.01	1.25	0.66	-4.11	-2.86
H(13A)	-C(13)	0.4182	0.666	1.89	-22.79	0.01	1.25	0.66	-4.1	-2.85
H(13B)	-C(13)	0.4187	0.6668	1.88	-22.72	0.01	1.25	0.66	-4.09	-2.84
H(13C)	-C(13)	0.4181	0.6653	1.89	-22.86	0.01	1.25	0.66	-4.11	-2.86
H(14A)	-C(14)	0.4185	0.6664	1.88	-22.76	0.01	1.25	0.66	-4.09	-2.84
H(14B)	-C(14)	0.4181	0.6661	1.89	-22.78	0.01	1.26	0.66	-4.1	-2.85
H(14C)	-C(14)	0.4181	0.6652	1.89	-22.88	0.01	1.25	0.66	-4.11	-2.86
C(15)	-C(16)	0.6939	0.6993	2.16	-20.52	0.22	1.95	0.9	-5.34	-3.39
C(15)	-C(20)	0.6901	0.6976	2.19	-20.81	0.23	1.99	0.91	-5.43	-3.44
H(16)	-C(16)	0.3754	0.6954	2	-24.13	0.03	1.42	0.71	-4.54	-3.11
H(18)	-C(18)	0.3769	0.697	2	-24.28	0.03	1.41	0.71	-4.52	-3.11
H(20)	-C(20)	0.376	0.6986	2	-24.4	0.04	1.42	0.71	-4.55	-3.13
C(17)	-C(16)	0.6911	0.695	2.2	-21.66	0.21	1.97	0.9	-5.46	-3.49
H(17)	-C(17)	0.3782	0.6957	1.99	-23.97	0.04	1.41	0.71	-4.49	-3.08
H(19)	-C(19)	0.3782	0.6955	1.98	-23.58	0.03	1.41	0.71	-4.47	-3.06
C(17)	-C(18)	0.6933	0.7005	2.17	-20.75	0.21	1.96	0.9	-5.38	-3.41
C(19)	-C(18)	0.6924	0.6955	2.2	-21.72	0.22	1.99	0.9	-5.5	-3.51
C(19)	-C(20)	0.6943	0.698	2.19	-21.4	0.23	1.98	0.9	-5.45	-3.48
C(21)	-C(22)	0.763	0.7665	1.73	-16.5	0.03	1.23	0.71	-3.61	-2.38
C(21)	-C(23)	0.7653	0.7697	1.71	-16.17	0.03	1.21	0.71	-3.55	-2.34
C(21)	-C(24)	0.7658	0.77	1.71	-16.22	0.03	1.21	0.71	-3.55	-2.34
H(22A)	-C(22)	0.4182	0.6652	1.89	-22.86	0.01	1.25	0.66	-4.11	-2.85
H(22B)	-C(22)	0.418	0.6653	1.89	-22.87	0.01	1.26	0.66	-4.11	-2.86
H(22C)	-C(22)	0.4181	0.6667	1.89	-22.76	0.01	1.26	0.67	-4.1	-2.85
H(23A)	-C(23)	0.4181	0.6661	1.89	-22.78	0.01	1.26	0.67	-4.11	-2.85
H(23B)	-C(23)	0.4181	0.6651	1.89	-22.88	0.01	1.25	0.66	-4.11	-2.86
H(23C)	-C(23)	0.4186	0.6663	1.88	-22.76	0.01	1.25	0.66	-4.09	-2.84
H(24A)	-C(24)	0.4183	0.666	1.89	-22.79	0.01	1.25	0.66	-4.1	-2.85

H(24B) -C(24)	0.418	0.6654	1.89	-22.86	0.01	1.26	0.66	-4.11	-2.86
H(24C) -C(24)	0.4187	0.6668	1.88	-22.72	0.01	1.25	0.66	-4.09	-2.84
C(25) -C(26)	0.763	0.7665	1.73	-16.5	0.03	1.23	0.71	-3.61	-2.38
C(25) -C(27)	0.7653	0.7697	1.71	-16.21	0.03	1.21	0.71	-3.55	-2.34
C(25) -C(28)	0.7658	0.7701	1.71	-16.18	0.03	1.21	0.71	-3.55	-2.34
H(26A) -C(26)	0.418	0.6666	1.89	-22.77	0.01	1.26	0.67	-4.11	-2.85
H(26B) -C(26)	0.4184	0.6661	1.89	-22.79	0.01	1.25	0.66	-4.1	-2.85
H(26C) -C(26)	0.4182	0.6654	1.89	-22.85	0.01	1.25	0.66	-4.11	-2.85
H(27A) -C(27)	0.4181	0.6653	1.89	-22.87	0.01	1.25	0.66	-4.11	-2.85
H(27B) -C(27)	0.4186	0.6666	1.88	-22.74	0.01	1.25	0.66	-4.09	-2.84
H(27C) -C(27)	0.418	0.6656	1.89	-22.82	0.01	1.26	0.66	-4.11	-2.85
H(28A) -C(28)	0.4186	0.6666	1.88	-22.74	0.01	1.25	0.66	-4.09	-2.84
H(28B) -C(28)	0.4181	0.6658	1.89	-22.81	0.01	1.25	0.66	-4.11	-2.85
H(28C) -C(28)	0.4181	0.6654	1.89	-22.86	0.01	1.25	0.66	-4.11	-2.85

Table S21. BCP properties of **2_Co**

A - B	d1	d2	f	del2f	ell	Gb	Gb/f	Vb	Eb
CO(1) -N(1)	1.0271	1.0814	0.44	7.46	0.04	0.55	1.26	-0.58	-0.03
CO(1) -N(2)	1.0328	1.0858	0.43	7.21	0.02	0.53	1.24	-0.56	-0.03
CO(1) -N(3)	1.0327	1.0855	0.43	7.22	0.02	0.53	1.24	-0.56	-0.03
CO(1) -N(4)	1.0271	1.0812	0.44	7.46	0.04	0.55	1.26	-0.58	-0.03
S(1) -N(1)	0.751	0.8453	1.81	-15.02	0.11	1.46	0.81	-3.97	-2.51
S(1) -N(2)	0.7555	0.8442	1.8	-14.61	0.12	1.46	0.81	-3.95	-2.49
C(1) -S(1)	0.8549	0.9349	1.39	-7.44	0.03	1.04	0.75	-2.6	-1.56
S(2) -N(3)	0.7554	0.8442	1.8	-14.62	0.12	1.46	0.81	-3.95	-2.49
S(2) -N(4)	0.751	0.8453	1.81	-15.02	0.11	1.46	0.81	-3.97	-2.51
C(13) -S(2)	0.8549	0.9349	1.39	-7.43	0.03	1.04	0.75	-2.6	-1.56
SI(1) -N(1)	0.7098	1.0167	0.95	6.49	0.03	1.04	1.1	-1.63	-0.59
SI(1) -C(7)	0.7435	1.1368	0.89	-0.25	0.02	0.65	0.73	-1.31	-0.67
SI(1) -C(8)	0.7447	1.1399	0.88	-0.36	0.02	0.64	0.72	-1.3	-0.66
SI(1) -C(9)	0.745	1.1409	0.88	-0.39	0.02	0.63	0.72	-1.3	-0.66
SI(2) -N(2)	0.7101	1.0185	0.95	6.4	0.03	1.03	1.09	-1.62	-0.59
SI(2) -C(10)	0.7434	1.1365	0.89	-0.24	0.02	0.65	0.73	-1.31	-0.67
SI(2) -C(11)	0.7449	1.1402	0.88	-0.36	0.02	0.64	0.72	-1.3	-0.66
SI(2) -C(12)	0.7449	1.1406	0.88	-0.38	0.02	0.64	0.72	-1.3	-0.66
SI(3) -N(3)	0.7101	1.0183	0.95	6.4	0.03	1.03	1.09	-1.62	-0.59
SI(3) -C(19)	0.7448	1.1401	0.88	-0.36	0.02	0.64	0.72	-1.3	-0.66
SI(3) -C(20)	0.745	1.1406	0.88	-0.38	0.02	0.63	0.72	-1.3	-0.66
SI(3) -C(21)	0.7434	1.1364	0.89	-0.23	0.02	0.65	0.73	-1.31	-0.67
SI(4) -N(4)	0.7098	1.0168	0.95	6.48	0.03	1.04	1.1	-1.62	-0.59
SI(4) -C(22)	0.7446	1.1398	0.88	-0.35	0.02	0.64	0.72	-1.3	-0.66
SI(4) -C(23)	0.7451	1.1409	0.88	-0.39	0.02	0.63	0.72	-1.3	-0.66
SI(4) -C(24)	0.7436	1.1369	0.89	-0.25	0.02	0.65	0.73	-1.31	-0.67
C(1) -C(2)	0.693	0.7002	2.16	-20.27	0.23	1.96	0.91	-5.34	-3.38
C(1) -C(6)	0.691	0.6966	2.19	-20.86	0.23	1.99	0.91	-5.43	-3.45
H(2) -C(2)	0.3782	0.6929	2	-24.5	0.04	1.41	0.71	-4.54	-3.13
H(4) -C(4)	0.3802	0.6934	1.99	-24.31	0.02	1.4	0.7	-4.5	-3.1

H(6)	-C(6)	0.3794	0.6949	1.99	-24.22	0.04	1.4	0.7	-4.5	-3.1
C(3)	-C(2)	0.6884	0.697	2.19	-21.38	0.23	1.98	0.9	-5.45	-3.47
H(3)	-C(3)	0.3802	0.6934	1.98	-23.69	0.03	1.4	0.71	-4.46	-3.06
H(5)	-C(5)	0.3803	0.6933	1.98	-23.71	0.03	1.4	0.71	-4.46	-3.06
H(2)	-H(21B)	1.2383	1.4227	0.03	0.29	0.64	0.02	0.6	-0.01	0.01
C(3)	-C(4)	0.6909	0.703	2.17	-20.74	0.23	1.96	0.9	-5.37	-3.41
C(5)	-C(4)	0.6875	0.6998	2.19	-21.28	0.23	1.98	0.9	-5.45	-3.47
C(5)	-C(6)	0.6919	0.7009	2.17	-20.76	0.22	1.96	0.9	-5.37	-3.41
H(7A)	-C(7)	0.4235	0.6631	1.82	-20.95	0.05	1.21	0.66	-3.89	-2.68
H(7B)	-C(7)	0.4236	0.6622	1.82	-21.01	0.04	1.21	0.66	-3.89	-2.68
H(7C)	-C(7)	0.4235	0.6617	1.83	-21.05	0.04	1.21	0.66	-3.9	-2.68
H(8A)	-C(8)	0.4239	0.6632	1.82	-20.92	0.05	1.21	0.66	-3.88	-2.67
H(8B)	-C(8)	0.4236	0.6621	1.83	-21.01	0.04	1.21	0.66	-3.89	-2.68
H(8C)	-C(8)	0.4236	0.6625	1.82	-20.99	0.05	1.21	0.66	-3.89	-2.68
H(9A)	-C(9)	0.4236	0.6622	1.82	-21.01	0.05	1.21	0.66	-3.89	-2.68
H(9B)	-C(9)	0.4237	0.6625	1.82	-20.97	0.04	1.21	0.66	-3.89	-2.68
H(9C)	-C(9)	0.4238	0.6631	1.82	-20.94	0.05	1.21	0.66	-3.88	-2.67
H(24C)	-H(9A)	1.5719	1.5811	0.01	0.09	4.33	0	0.49	0	0
H(10A)	-C(10)	0.4236	0.6618	1.83	-21.04	0.04	1.21	0.66	-3.89	-2.68
H(10B)	-C(10)	0.4237	0.6626	1.82	-20.97	0.05	1.21	0.66	-3.89	-2.68
H(10C)	-C(10)	0.4235	0.663	1.82	-20.95	0.05	1.21	0.66	-3.89	-2.68
H(14)	-H(10B)	1.239	1.4254	0.03	0.29	0.64	0.02	0.6	-0.01	0.01
H(11A)	-C(11)	0.4236	0.6622	1.82	-21	0.04	1.21	0.66	-3.89	-2.68
H(11B)	-C(11)	0.4239	0.6633	1.82	-20.92	0.05	1.21	0.66	-3.88	-2.67
H(11C)	-C(11)	0.4236	0.6622	1.82	-21.01	0.05	1.21	0.66	-3.89	-2.68
H(12A)	-C(12)	0.4236	0.6622	1.83	-21.01	0.05	1.21	0.66	-3.89	-2.68
H(12B)	-C(12)	0.4238	0.663	1.82	-20.94	0.05	1.21	0.66	-3.88	-2.67
H(12C)	-C(12)	0.4236	0.6625	1.82	-20.98	0.04	1.21	0.66	-3.89	-2.68
H(12A)	-H(24B)	1.3794	1.4082	0.02	0.14	0.2	0.01	0.42	-0.01	0
C(13)	-C(14)	0.693	0.7002	2.16	-20.26	0.23	1.96	0.91	-5.34	-3.38
C(13)	-C(18)	0.6909	0.6965	2.19	-20.86	0.23	1.99	0.91	-5.44	-3.45
H(14)	-C(14)	0.3782	0.6929	2	-24.5	0.04	1.41	0.71	-4.54	-3.13
H(16)	-C(16)	0.3802	0.6934	1.99	-24.31	0.02	1.4	0.7	-4.5	-3.1
H(18)	-C(18)	0.3794	0.6949	1.99	-24.22	0.04	1.4	0.7	-4.5	-3.1
C(15)	-C(14)	0.6884	0.697	2.19	-21.39	0.23	1.98	0.9	-5.45	-3.47
H(15)	-C(15)	0.3802	0.6934	1.98	-23.69	0.03	1.4	0.71	-4.46	-3.06
H(17)	-C(17)	0.3803	0.6933	1.98	-23.71	0.03	1.4	0.71	-4.46	-3.06
C(15)	-C(16)	0.6909	0.703	2.17	-20.74	0.23	1.96	0.9	-5.37	-3.41
C(17)	-C(16)	0.6875	0.6998	2.19	-21.28	0.23	1.98	0.9	-5.45	-3.47
C(17)	-C(18)	0.6919	0.7009	2.17	-20.75	0.22	1.96	0.9	-5.37	-3.41
H(19A)	-C(19)	0.4238	0.6632	1.82	-20.92	0.05	1.21	0.66	-3.88	-2.67
H(19B)	-C(19)	0.4236	0.6623	1.83	-21	0.05	1.21	0.66	-3.89	-2.68
H(19C)	-C(19)	0.4236	0.6622	1.82	-21	0.04	1.21	0.66	-3.89	-2.68
H(20A)	-C(20)	0.4236	0.6621	1.82	-21.01	0.05	1.21	0.66	-3.89	-2.68
H(20B)	-C(20)	0.4238	0.663	1.82	-20.94	0.05	1.21	0.66	-3.88	-2.67
H(20C)	-C(20)	0.4236	0.6625	1.82	-20.98	0.05	1.21	0.66	-3.89	-2.68
H(21A)	-C(21)	0.4236	0.6618	1.83	-21.04	0.04	1.21	0.66	-3.89	-2.68

H(21B)	-C(21)	0.4237	0.6626	1.82	-20.97	0.05	1.21	0.66	-3.89	-2.68
H(21C)	-C(21)	0.4235	0.663	1.82	-20.95	0.05	1.21	0.66	-3.89	-2.68
H(22A)	-C(22)	0.4236	0.6621	1.82	-21.01	0.04	1.21	0.66	-3.89	-2.68
H(22B)	-C(22)	0.4236	0.6625	1.82	-20.98	0.05	1.21	0.66	-3.89	-2.68
H(22C)	-C(22)	0.4239	0.6632	1.82	-20.92	0.05	1.21	0.66	-3.88	-2.67
H(7C)	-H(23B)	1.5851	1.6033	0.01	0.09	30.69	0	0.48	0	0
H(23A)	-C(23)	0.4238	0.663	1.82	-20.94	0.05	1.21	0.66	-3.88	-2.67
H(23B)	-C(23)	0.4237	0.6622	1.82	-21.01	0.05	1.21	0.66	-3.89	-2.68
H(23C)	-C(23)	0.4237	0.6626	1.82	-20.97	0.04	1.21	0.66	-3.89	-2.68
H(24A)	-C(24)	0.4235	0.663	1.82	-20.95	0.05	1.21	0.66	-3.89	-2.68
H(24B)	-C(24)	0.4236	0.6622	1.82	-21.01	0.04	1.21	0.66	-3.89	-2.68
H(24C)	-C(24)	0.4235	0.6618	1.83	-21.05	0.04	1.21	0.66	-3.9	-2.68

Literaturstellen

[54]O. Kahn, Molecular Magnetism, VCH Publishers Inc., New York, 1993.

[55]E. Bill, julX_2s, Simulation of molecular magnetic data software, Max-Planck Institute for Bioinorganic Chemistry: Mülheim, Ruhr, **2014**

[56]Guo, Y.-N.; Xu, G.-F.; Guo, Y.; Tang, J. “Relaxation dynamics of dysprosium(III) single molecule magnets” *Dalton Trans.* **2011,** *40* (39), 9953–9963.

[57] Reta, D.; Chilton, N. F. “Uncertainty estimates for magnetic relaxation times and magnetic relaxation parameters” *Phys. Chem. Chem. Phys.* **2019,** *21* (42), 23567–23575

[58]D. Gatteschi, R. Sessoli, “Quantum Tunneling of Magnetization and Related Phenomena in Molecular Materials“ *Angew. Chem. Int. Ed.* **2003**, *42*, 268-297.

[59] K. N. Shrivastava, “Theory of Spin–Lattice Relaxation” *phys. stat. sol. (b)* **1983**, *117*, 437–458.

[60]R. Orbach, “Spin-lattice relaxation in rare-earth salts” *Proc. R. Soc. Lond. A* **1961**, *264*, 458–484.

[61]OriginPro, Version 2020. OriginLab Corporation, Northampton, MA, USA.

[62]L Krause, B. Niepötter, C. J. Schürmann, D.Stalke, R. Herbst-Irmer, “Validation of experimental charge-density refinement strategies: when do we overfit?” *IUCrJ*, **2017**, *4*, 420–430

[63] S. C. Abrahams, E. T. Keve, “Normal Probability Plot Analysis of Error in Measured and Derived Quantifies and Standard Deviations” *Acta Crystallogr.* **1971**, *A27*, 157–165.

[64]A. Stash. *DRKplot*, Moscow (Russia) **2007**

[65]K. Meindl, J. Henn, “Foundations of residual-density analysis” *Acta Crystallogr.*, **2008**, A*64*, 404-418.
